# Total Reversal of ALS Confirmed by EMG Normalization, Structural Reconstitution, and Neuromuscular–Molecular Restoration Achieved Through Computerized Brain-Guided Reengineering of the 1927 Nobel Prize Fever Therapy: A Case Report

**DOI:** 10.3390/diseases13110371

**Published:** 2025-11-12

**Authors:** M. Marc Abreu, Mohammad Hosseine-Farid, David G. Silverman

**Affiliations:** 1 Clinical Sciences Division, BTT Medical Institute, Aventura, FL 33160, USA; 2 Department of Biomedical Engineering and Medical Physics, BTT Medical Institute, Aventura, FL 33180, USA; 3 College of Computing and Engineering, Nova Southeastern University, Fort Lauderdale, FL 33314, USA; mfarid@nova.edu; 4 Department of Anesthesiology, Yale University School of Medicine, New Haven, CT 06510, USA

**Keywords:** amyotrophic lateral sclerosis, ALS treatment, ALS, computerized brain-guided intelligent thermofebrile therapy, heat shock protein, brain–eyelid thermoregulatory tunnel, fever therapy, neurodegeneration reversal, Nobel prize-recognized malarial fever therapy

## Abstract

Background: Neurological disorders are the leading cause of disability, affecting over three billion people worldwide. Amyotrophic lateral sclerosis (ALS) is among the most feared and uniformly fatal neurodegenerative diseases, with no therapy capable of restoring lost function. Methods: We report the first application of therapeutic fever to ALS using Computerized Brain-Guided Intelligent Thermofebrile Therapy (CBIT^2^). This fully noninvasive treatment, delivered through an FDA-approved computerized platform, digitally reengineers the 1927 Nobel Prize-recognized malarial fever therapy into a modern treatment guided by the Brain–Eyelid Thermoregulatory Tunnel. CBIT^2^ induces therapeutic fever through synchronized hypothalamic feedback, activating heat shock proteins, which are known to restore proteostasis and neuronal function. Case presentation: A 56-year-old woman was diagnosed with progressive ALS at the Mayo Clinic, with electromyography (EMG) demonstrating fibrillation and fasciculation indicative of denervation corroborated by neurological and MRI findings; the patient was informed that she had an expected survival of three to five years. A neurologist from Northwestern University confirmed the diagnosis and thus maintained the patient on FDA-approved ALS drugs (riluzole and edaravone). Her condition rapidly worsened despite pharmacological treatment, and she underwent CBIT^2^, resulting in (i) electrophysiological reversal with complete disappearance of denervation; (ii) biomarker correction, including reductions in neurofilament and homocysteine, IL-10 normalization (previously linked to mortality), and robust HSP70 induction; (iii) restoration of gait, swallowing, respiration, speech, and cognition; (iv) reconstitution of tongue structure; and (v) return to complex motor tasks, including golf, pickleball, and swimming. Discussion: This case provides the first documented evidence that ALS can be reversed through digitally reengineered fever therapy aligned with thermoregulation, which induces heat shock response and upregulates heat shock proteins, resulting in the patient no longer meeting diagnostic criteria for ALS and discontinuation of ALS-specific medications. Beyond ALS, shared protein-misfolding pathology suggests that CBIT^2^ may extend to Alzheimer’s, Parkinson’s, and related disorders. By modernizing this Nobel Prize-recognized therapeutic principle with computerized precision, CBIT^2^ establishes a framework for large-scale clinical trials. A century after fever therapy restored lost brain function and so decisively reversed dementia paralytica such that it earned the 1927 Nobel Prize in Medicine, CBIT^2^ now safely harnesses the therapeutic power of fever through noninvasive, intelligent, brain-guided thermal modulation. Amid a global brain health crisis, fever-based therapies may offer a path to preserve thought, memory, movement, and independence for the more than one-third of humanity currently affected by neurological disorders.

## 1. Introduction

“We have an urgent global brain health crisis… No country has a handle on this escalating challenge” [1]. With these words, the June 2025 G7 Summit of the world’s leading economies highlighted that even the most technologically advanced nations, equipped with cutting-edge technology and pharmaceuticals, remain unable to contain the accelerating neurological epidemic [1]. The G7 declaration echoed the World Health Organization (WHO)’s warning in 2024 that neurological disorders now affect more than one in three people, totaling over three billion individuals, and have become the leading cause of disability worldwide [2,3]; this reflects a lack of treatments capable of restoring lost neurological function.

In the absence of therapies capable of reversing the disease, neurological disorders will not only cause disability but also lead to death. This loss of human life is no longer theoretical, as the WHO has issued a warning that neurological disorders are projected to become the second leading cause of death worldwide [3,4], confirming that the present crisis of disability is rapidly escalating into a global mass mortality event resulting in substantial socioeconomic consequences.

Among the most feared and devastating manifestations of neurological disease is amyotrophic lateral sclerosis (ALS), a progressive, fatal neurodegenerative disorder characterized by paralysis [5,6] and often dementia [7,8]; it strips away the most basic human abilities such as moving, speaking, swallowing, and breathing. ALS gained widespread recognition following the death of New York Yankees Hall of Fame baseball star Lou Gehrig at age 37, demonstrating that neither youth, peak physical fitness, nor elite athletic performance confers protection against the disease’s indiscriminate nature [9,10].

Long considered untreatable, ALS progresses to paralysis, respiratory failure, and death. Confronting this challenge demands not only new treatments but also an entirely new way of thinking. However, the most transformative idea may not be new at all, but one discovered a century ago in the form of malarial fever therapy, which effectively restored neurological function in dementia paralytica and was honored with the Nobel Prize in Medicine in 1927 [11,12,13,14,15,16,17,18,19,20]. This suggests that the principles underlying fever therapy may warrant renewed investigation in the modern medical era.

The convergence of a Nobel Prize-recognized treatment and advanced digital engineering has unlocked a novel approach, reviving a long-overlooked path through a scientifically grounded method that, in this case, achieved the neurological, molecular, anatomical, and electrophysiological reversal of ALS, opening the path to treating a wide range of neurodegenerative diseases previously deemed irreversible. This avenue for effectively treating neurological disorders was made possible by the approval of a computerized platform by the U.S. Food and Drug Administration (FDA), publicly announced by Asus Computer Company [21], which enabled the development of Computerized Brain-Guided Intelligent Thermofebrile Therapy (CBIT^2^). This approach digitally reengineers the 1927 Nobel Prize-winning malarial fever therapy that once achieved the unthinkable by reversing paralysis and dementia in patients neurologically condemned to death [11,12,13,14,15,16,17,18,19,20].

To counteract ongoing neuronal loss and progressive incapacitation in a 56-year-old patient with rapidly advancing ALS, despite treatment with FDA-approved ALS medications, we used CBIT^2^, a digitally controlled, fully noninvasive, intelligent, noninfectious, fever-based therapy designed to regulate the brain’s thermoregulatory response and activate cerebral molecular heat shock repair systems via heat shock response. Using digital precision and real-time thermoregulatory feedback, CBIT^2^ was employed to induce heat shock protein (HSP) in the brain, specifically targeting motor neurons, with the aim of counteracting a biomarker-confirmed trajectory of rapid disease progression and impending demise in the patient while reversing neurodegeneration and overcoming the well-documented limitations of existing ALS therapies. Current ALS treatments offer only marginal delays in functional decline and brief extensions of life by about two to three months [22,23], but they fail to arrest or reverse neuronal loss, progressive incapacitation, and death.

This report emerges in the context of a mounting global neurological crisis affecting over one in three people worldwide [1,2]. Paradoxically, it is ALS, which has long been considered an intractable and fatal neurodegenerative disorder, that may offer a path towards responding to the global neurological emergency. Given ALS’s uniquely high threshold for inducing HSPs in motor neurons [24], the remarkable combination of neurological, structural, molecular, and electrophysiological reversal observed in this ALS patient suggests a broader therapeutic potential that extends well beyond a single neurological disease, as neurological disorders share common features of progressive neuronal loss driven by pathological protein aggregation [25,26]. Among these disorders, ALS may serve as the ultimate proving ground; if reversal is achievable in this most treatment-resistant condition [24], then applying a similar therapeutic approach to less refractory neurological diseases, such as Alzheimer’s disease, Parkinson’s disease, ataxia, and related pathologies, may not only be plausible but imminently within reach. Thus, ALS shifts from a symbol of irreversible decline and death to a therapeutic gateway, offering a path forward in the broader fight against neurological disorders.

This case report reclaims a therapeutic truth first revealed through the Nobel Prize-winning malarial fever treatment, which is the only treatment in history to cure dementia paralytica and empty the asylums once filled with individuals terminally afflicted with this fatal neuropsychiatric disorder [11,12,13,14,15,16,17,18,19,20]. This report opens the door to reconsidering fever as a therapy potentially capable of restoring proteostasis. Induction of HSPs has been shown to promote refolding and clearance of misfolded proteins such as TDP-43 and to reduce their associated toxicity, which has been consistently demonstrated in numerous experimental investigations [27,28,29,30,31], offering a path to protect and restore neurological health.

In the case report presented herein, effective heat shock response with HSP induction after computerized fever-based therapy led to the neurological, molecular, structural and electrophysiological reversal of ALS. This therapeutic rationale is strengthened by the fact that the molecular basis of neurodegeneration is impaired HSP function, which leads to misfolded protein accumulation, neuronal damage, and disease progression [32,33,34]. Furthermore, increased levels of HSP70 reduce protein aggregation and support motor neuron survival [35,36], while HSP27 and HSP90 influence neuroprotection or disease progression based on their expression levels [37,38]. HSP-based pharmacological interventions have been considered for Alzheimer’s, Parkinson’s, Huntington’s, and ALS [39,40,41,42,43], with the aim of correcting chaperone dysfunction, restoring proteostasis [44]. Recent attempts to upregulate HSPs using agents such as arimoclomol showed increased HSP expression in animal models but failed to improve clinical outcomes and raised safety concerns at higher doses [45]. Nonetheless, HSP activation may hold therapeutic and neuroprotective potential, as detailed in a recent review by Smadja and Abreu [26] and emerging evidence suggests that passive heat therapy may induce HSP expression and reduce the risk of Alzheimer’s and Parkinson’s disease [46]. However, although a relationship between hyperthermia and HSP activation has been established [26], thermal treatment has never been applied to ALS, and its use to counter, or even reverse, the associated paralysis and cognitive decline has not yet been explored.

We herein demonstrate the therapeutic effects of CBIT^2^ in ALS, with evidence of disease reversal and restoration of motor neuron function, as validated by objective neuromuscular assessments, normalization of molecular biomarker, reconstitution of tongue structure, and upregulation of HSP expression. Remarkably, electrophysiological studies revealed the complete disappearance of denervation, the defining hallmark of motor neuron death in ALS, along with the resolution of fasciculations; both were clearly present before our treatment but absent following CBIT^2^. This electromyographic reversal of ALS following CBIT^2^ was independently validated at one of the foremost university-based neurology centers in the United States [for an electromyography (EMG) report, see Appendix A], providing rigorous objective evidence for the neurodegeneration reversal in a condition long considered irreversible. What was once deemed impossible in ALS occurred following CBIT^2^, as EMG demonstrated complete disappearance of fibrillation and fasciculation, providing objective evidence for the absence of lower motor neuron degeneration. Thus, the patient no longer meets the diagnostic criteria for ALS post treatment with CBIT^2^.

As noted earlier, the protocol used in CBIT^2^ builds on a landmark discovery in medical history, when Austrian psychiatrist Dr. Julius Wagner-Jauregg pioneered the use of high fever induced by malarial infection to effectively treat dementia paralytica, a disorder marked by fatal paralysis and cognitive decline in the terminal stage of neurosyphilis, for which he was awarded the Nobel Prize in Medicine [11,12,13,14,15,16,17,18,19,20]. As one review described, the impact of malarial fever therapy was dramatic: “Death, in most cases, was welcomed as the final respite from the horrifying symptoms of neurosyphilis… malarial treatment played a role in the emptying of the asylums and provided a viable alternative for a previously hopeless disease” [12].

Dr. Wagner-Jauregg initially used erysipelas and tuberculin to induce therapeutic fever, with limited success and severe side effects. His breakthrough came with the use of malarial fever, which resulted in remarkable neurological recovery in patients with dementia paralytica, ultimately establishing the treatment as a curative therapy for otherwise intractable neuropsychiatric symptoms [11,12,13,14,15,16,17,18,19,20]. At the time, the therapeutic benefit was primarily attributed to fever’s presumed ability to eliminate *Treponema pallidum*, the pathogen causing syphilis, rather than reversal of brain disease, which is the therapeutic paradigm newly proposed here. As further evidence supporting bacterial eradication as the therapeutic rationale a century ago, some physicians advocated initiating malarial fever therapy immediately following a positive Wassermann test, even before the onset of dementia paralytica, as a preventive intervention against neurosyphilis [12]. Despite its remarkable success in Europe and across the world, malarial fever therapy faded into obscurity with the advent of antibiotics (which were used to prevent the progression to neurosyphilis) and the risk of parasite migration to the brain and subsequent cerebral malaria, which is commonly fatal [11,12,13,14,15,16,17,18,19,20].

Nearly a century later, M. Marc Abreu, M.D. (primary author) critically re-examined Wagner-Jauregg’s original data and uncovered a striking and long-overlooked possibility that malarial fever therapy may have reversed not only the underlying syphilitic infection but also the structural brain injury that persisted even after microbial eradication. Abreu observed that the progressive paralysis seen in dementia paralytica closely resembles the motor deterioration characteristic of ALS. Remarkably, these two seemingly unrelated diseases are united by a shared molecular signature: both are driven by the pathological accumulation of misfolded TDP-43, the defining biomarker of ALS [47], which, surprisingly, also aggregates in neurosyphilis [48]. This unexpected molecular signature offers compelling scientific evidence for hypothesizing that the curative principles behind the 1927 Nobel Prize-winning therapy may hold similar promise for effectively treating ALS today.

Molecular evidence implicates misfolded TDP-43 as a central driver of neurodegeneration [27,28,29,30,31], which in combination with therapeutic fever from tertian malaria reaching up to 41.6 °C [11,12,13,14,15,16,17,18,19,20,49,50,51,52] enabled full neurological recovery with reintegration of patients into daily life [11,12,13,14,15,16,17,18,19,20], demonstrating that neurological damage can, in fact, be reversed. Multiple reports from around the world describe patients once bound by paralysis and seemingly condemned to death who, after malarial fever therapy, regained speech, movement, and functional independence, outcomes regarded as neurological rebirth [11,12,13,14,15,16,17,18,19,20]. Building on the extraordinary outcomes and recognizing the need for a safe, effective, and noninfectious alternative, Abreu developed a computerized, digitally controlled, intelligent, hypothalamus-guided platform designed to replicate the high fever cyclical patterns of malarial infection [49,50,51,52] to induce heat shock response, which is known to facilitate clearance of misfolded TDP-43 in experimental model of ALS [27,28]. The brain-guided fever therapy introduced here seeks to reverse neurodegeneration previously deemed irreversible, drawing on the established role of the heat shock response in promoting TDP-43 clearance, toxicity reduction, and neuronal protection [27,28,29,30,31].

The safe titration of brain temperature and the generation of computerized cyclical thermal patterns under hypothalamic control were made possible by Abreu’s discovery at the Yale University School of Medicine of a biological thermal waveguide between the brain and eyelid, known as the Brain–Eyelid Thermoregulatory Tunnel (BTT), which was initially described as the brain temperature tunnel [53,54,55]. The discovery of the BTT led to the development of a computerized platform and sensor system approved by the U.S. FDA, which laid the technological foundation for CBIT^2^. This system offers noninvasive measurements of brain temperature and the real-time, brain-guided, intelligent induction of the heat shock response (Abreu BTT 700 Computer System, Heat Shock Induction 700 Module, Brain Tunnelgenix Technologies Corp., Aventura, Florida, USA). Agreement with body core temperature, except for specificity to the brain during brain–core discordance, was demonstrated in Yale-University-led studies, conducted in New Haven, Connecticut, USA, by Abreu and Silverman (co-author) in collaboration with other investigators and institutions [55], as well as in a multi-institutional study led by Nova Southeastern University in Florida, USA, which cited the Yale findings in an article assessing the impact of carotid temperature modifications on brain temperature and yawning [56].

The misfolded protein pathology shared by neurosyphilis [48] and ALS [47], together with worldwide clinical evidence of fever-induced neurological recovery [11,12,13,14,15,16,17,18,19,20], provided the conceptual basis for CBIT^2^, a computerized, noninvasive, hypothalamus-targeted, artificial intelligence (AI)-enhanced platform designed to titrate brain temperature and aimed to induce heat shock response to reverse neurodegeneration. Consistent with the rhythmic nature of malarial fever [49,50,51,52], Abreu hypothesized that such patterned thermal input triggers repeated activation of the heat shock response, upregulating HSPs that support neuronal protection and recovery [32,33,34,35,36,37,44,57,58], and further proposed that cerebral HSP induction may respond more effectively to dynamic thermal profiles across distinct brain regions [59].

Noninvasive brain temperature and thermodynamics monitoring are critical to both the safety and efficacy of CBIT^2^, enabling the real-time assessment of cerebral thermodynamics, precise titration of the thermofebrile response based on hypothalamic signals, prevention of hyperthermic brain injury, and direct delivery of therapeutic heat to the brain. These advantages are especially critical in ALS, in which motor neurons require a higher thermal threshold to induce heat shock response, including robust expression of HSP70 [24]. Guided by the BTT, CBIT^2^ delivers precise, cyclical high heat exposure to activate HSPs within thermally resistant motor neurons [24], replicating the therapeutic mechanism of malarial fever [49,50,51,52] in a computerized, infection-free modality.

By implementing continuous temperature measurements for real-time adjustments, the protocol ensures precise brain thermal modulation and patient safety, reflecting Lord Kelvin’s principle that “You cannot manage what you cannot measure” [60]. Clinical outcomes were documented through enhanced motor function, reductions in pathological serum biomarkers, measurable increases in HSP expression, reconstitution of tongue structure, and electrophysiological restoration, supported by patient and physical therapist report. A unique strength of this case report is the integration of pre- and post-treatment video documentation, which provides direct visual validation of neurological recovery, corroborating findings from EMG and blood biomarkers. This convergence of objective clinical, molecular, and electrophysiological data with real-time video documentation confirms patient outcomes and reinforces the scientific rationale for CBIT^2^, which is based on the long-overlooked therapeutic principles of malarial fever therapy.

Recent evidence has further strengthened the therapeutic rationale for inducing the heat shock response in neurodegenerative diseases. Ahmed et al. demonstrated that amplifying the heat shock response markedly ameliorates both ALS and pathology in mouse and human models [27]. Activation of HSF1 led to upregulation of heat shock proteins such as HSP70 and HSP40, which promoted the refolding and clearance of misfolded TDP-43; this is the central pathogenic protein in ALS [47] and it is also found in neurosyphilis [48]. Heat shock response restores neuronal proteostasis, reduced cytoplasmic TDP-43 inclusions, improved neuronal survival, and preserved motor function [27,28,29,30,31]. These findings provide compelling mechanistic support for therapies that induce the heat shock response to counter neurodegeneration, forming part of the scientific foundation for the present treatment of ALS using CBIT^2^, a digitally controlled, thermoregulation-guided platform designed to activate the heat shock response through brain-guided noninfectious fever induction.

The forgotten or, more aptly, unrecognized and unappreciated link between the 1927 Nobel Prize discovery and the treatment of neurodegenerative disease laid the foundation for a safe, infection-free, computer-controlled hub of therapeutic intelligence capable of dynamically modulating and personalizing therapy in real time. By digitally reengineering the century-old, Nobel Prize-recognized malarial fever therapy [11,12,13,14,15,16,17,18,19,20] that once reversed disease and restored function in patients with paralysis and dementia, CBIT^2^ delivers precise, brain-guided replication of the cyclical thermal dynamics of malarial infection [49,50,51,52] to treat neurodegeneration.

Amid the escalating global brain health crisis highlighted by the G7 Summit and the WHO [1,2], the principles of this Nobel Prize-recognized fever therapy are reinterpreted through modern digital engineering to offer a potential therapeutic approach for conditions once deemed irreversible. Through AI-enhanced brain thermodynamics, the rhythmic thermal patterns once generated unpredictably by infection can now be delivered with precision, safety, and hypothalamic targeting, transforming an abandoned historical intervention into a controllable, noninvasive neurotherapeutic platform.

This case report documents the neurological, molecular, structural, and electrophysiological reversal of ALS following CBIT^2^, and plausibly links its therapeutic effect to the induction of the heat shock response targeting misfolded TDP-43, which is based on multiple experimental investigations showing that activation of the heat shock response leads to expression of HSPs, particularly HSP70 and HSP40, which have been shown to facilitate clearance and refolding of misfolded TDP-43 [27,28,29,30,31]. These findings support the hypothesis that, through AI-enhanced modulation of brain thermodynamics, CBIT^2^ replicates fever-induced activation of the heat shock response to counteract TDP-43-associated neurodegeneration. Enabled by the U.S. FDA approved computerized platform [21], CBIT^2^ transforms the century-old, Nobel-recognized principle of fever therapy for disease reversal into a modern, infection-free, brain-guided intervention capable of precise thermoregulatory control. By harnessing the activation of the heat shock response through digital brain thermodynamics, CBIT^2^ offers a plausible and scientifically grounded pathway to alter the course of neurological decline and reopen the long-dormant field of therapeutic fever.

### Case Report

A 56-year-old female patient with a confirmed diagnosis of ALS was referred to the BTT Medical Institute in Florida, USA, following a progressive and marked decline in motor function. On 25 October 2024, she was diagnosed with ALS at the Mayo Clinic in Rochester, Minnesota, USA, which is recognized as the leading neurology hospital in the United States [61].

Her past medical history was otherwise unremarkable, aside from a cervical fusion at the C3–C5 vertebrae without residual sequelae. Her family history was negative for dementia, neurological disorders, or muscle disease. She denied smoking or recreational drug use and reported social consumption of approximately one alcoholic drink per week. In May 2019, she developed mild stiffness and spasticity in both legs after a fall, which led to recurrent falls. Initially suspected of having Stiff Person Syndrome, she was treated with intravenous immunoglobulin (IVIg) and diazepam, but her symptoms failed to improve and progressively worsened. In September 2023, she developed dysphagia and dysarthria, which worsened in the evenings, despite a reduction in her diazepam dosage.

The cornerstone for diagnosing ALS is EMG showing denervation. EMG was performed at the Mayo Clinic on 25 October 2024, and it demonstrated active denervation and reinnervation in the lumbosacral segment with chronic denervation in the right hand, in addition to fasciculations demonstrating lower motor neuron changes (see Appendix A for full report). The Mayo Clinic report further documented progressive weakness of hand grip, which was more pronounced on the right side, along with progressive spasticity of the lower extremities and worsening gait; progression of disease was evidenced by the patient requiring a walker after previously using a cane. The patient also presented with dysarthria, episodes of coughing when drinking liquids, and nasal regurgitation, all of which were progressively worsening. On examination, her gait was markedly spastic; she was unable to walk on heels or toes and could not rise from a chair. Additional findings included a positive Hoffmann sign, weakness of the right arm and hand, and weakness involving the knee, ankle, and toes. Taken together, the findings further corroborate it, as seen in the changes in the EMG report (Appendix A.1), consistent with the diagnosis of ALS. The Mayo Clinic neurologist stressed in writing that “progressive lower motor neuron denervation is unfortunately inevitable” and that “The median patient survival is 3–5 years from symptom onset considering all ALS patients, but some patients have a more rapid or slowly progressive curve.” The patient was enrolled in the ALS Clinic and prescribed FDA-approved drugs for ALS, namely riluzole and edaravone.

MRI demonstrated susceptibility-weighted imaging (SWI) changes in the motor band and corticospinal tract (CST) hyperintensity on FLAIR (fluid-attenuated inversion recovery) in the pons and cerebellar peduncles. The combined presence of the motor band sign on SWI and corticospinal tract hyperintensity on FLAIR has been reported to carry high specificity for ALS, thereby providing additional radiologic corroboration of the ALS diagnosis. A series of tests and evaluations were performed to rule out alternative diagnoses, including Lyme serology and a long-chain fatty acid panel, the results of which were normal, and testing for myasthenia gravis, M protein, creatine kinase (CK), hemoglobin A1c, and thyroid-stimulating hormone (TSH), all of which were negative.

The patient’s diagnostic and therapeutic journey spanned two of the most prestigious neurology institutions in the United States. She was initially diagnosed and treated at the Mayo Clinic and later at Northwestern University, a nationally ranked neurology center in Illinois [61], where the patient resides. Together, these centers represent the pinnacle of neurological expertise, yet her rapidly declining course underscores the reality that even care delivered at the most elite institutions by some of the foremost neurologists in the field remains constrained by the limited efficacy of current ALS therapies. Her neurological function continued to deteriorate despite treatment with two FDA-approved therapies for ALS, which are known to provide only modest clinical benefit. This trajectory highlights not only the limitations of standard pharmacologic options but also the urgent need for alternative therapeutic strategies capable of reversing disease and restoring lost neurological function.

A neurological examination performed at Northwestern on 4 December 2024 documented mixed dysarthria and a spastic gait. Cranial nerve examination revealed a weak cheek puff and slow tongue movement. Motor examination revealed reduced motor strength and spasticity, predominantly in the right leg, as well as positive Romberg, Hoffman, and Tromner’s signs (which were more pronounced on the right). The patient had a limited range of motion but no bony abnormalities, contractures, malalignment, or tenderness. The diagnosis of ALS was confirmed, and the neurologist at Northwestern University maintained the patient on riluzole and edaravone, the only drugs exclusively approved by the U.S. FDA for ALS, thereby further reinforcing the diagnosis of ALS.

The patient learned about brain-guided programmed fever therapy from an acquaintance who had previously been successfully treated at the BTT Medical Institute. A pre-treatment examination performed at the BTT Medical Institute in Florida, USA, on 28 January 2025, confirmed findings consistent with prior evaluations at the Mayo Clinic and Northwestern University, which are detailed herein. However, by this time, the disease had progressed, and the patient’s gait had become irregular, wide-based, and waddling, with severely limited ambulation requiring the assistance of a walker. Coordination and cerebellar testing revealed intact finger-to-nose performance without tremors. Her current medications included diazepam 5 mg once daily by mouth, Sertraline 50 mg once daily by mouth, dextromethorphan/quinidine 10 mg twice daily by mouth, riluzole 50 mg twice daily by mouth, and edaravone 5 mL once daily by mouth. Ongoing and supportive management consisted of physical therapy, speech therapy, and symptomatic treatment aimed at managing spasticity, dysarthria, and dysphagia.

We sought to determine whether Computerized Brain-Guided Intelligent Thermofebrile Therapy (CBIT^2^), delivered through an FDA-approved computerized platform incorporating a Thermofebrile Rhythm Engineering Protocol, could acutely halt, or even reverse, the patient’s ongoing neurodegeneration and motor neuron loss due to ALS. This therapeutic application of CBIT^2^ in a patient with a neurodegenerative disorder was guided by the hypothesis that CBIT^2^ could reproduce the curative effects once achieved with malarial fever therapy, grounded in the pathological hallmark of TDP-43 proteinopathy, which is present in both ALS and dementia paralytica (neurosyphilis) [47,48]; this is the disease effectively treated by Wagner-Jauregg’s Nobel Prize-winning discovery. The mechanistic framework underlying this hypothesis linking fever-induced heat shock response to potential TDP-43 clearance and neuronal recovery is detailed in the Discussion.

To rigorously evaluate this potential therapeutic response, as part of our clinical care, we performed a comprehensive battery of assessments, including quantitative neuromuscular testing tailored to the patient’s symptom profile, high-resolution gait and balance analysis, respiratory function testing, oral motor strength evaluation, and upper and lower limb strength measurement. Serial blood biomarkers were monitored to assess disease burden and therapeutic response, including neurofilament light chain (NfL), homocysteine, interleukin-10 (IL-10), TNF, and HSP expression. Complementing these molecular assessments, electrophysiological studies, including pre- and post-treatment EMGs spaced five months apart, were conducted by independent university-based services to evaluate the presence of denervation and other EMG changes, such as reinnervation. In this way, the electrophysiological data provided a real-time demonstration of the absence of denervation following CBIT^2^, which is a crucial finding, since denervation represents the death of lower motor neurons, thereby reflecting underlying neuronal restoration objectively manifested within the affected muscles.

This multidimensional evaluation, including neuromuscular assessment, serial biomarkers, anatomical evaluation, and electrophysiological studies, enabled a robust appraisal of both functional and structural recovery and the molecular response to the treatment with CBIT^2^.

## 2. Therapeutic Intervention

### 2.1. Computerized, Intelligent Thermal Delivery via Radiative–Conductive Integration and Hypothalamic Feedback Modulation

**Figure 1:**
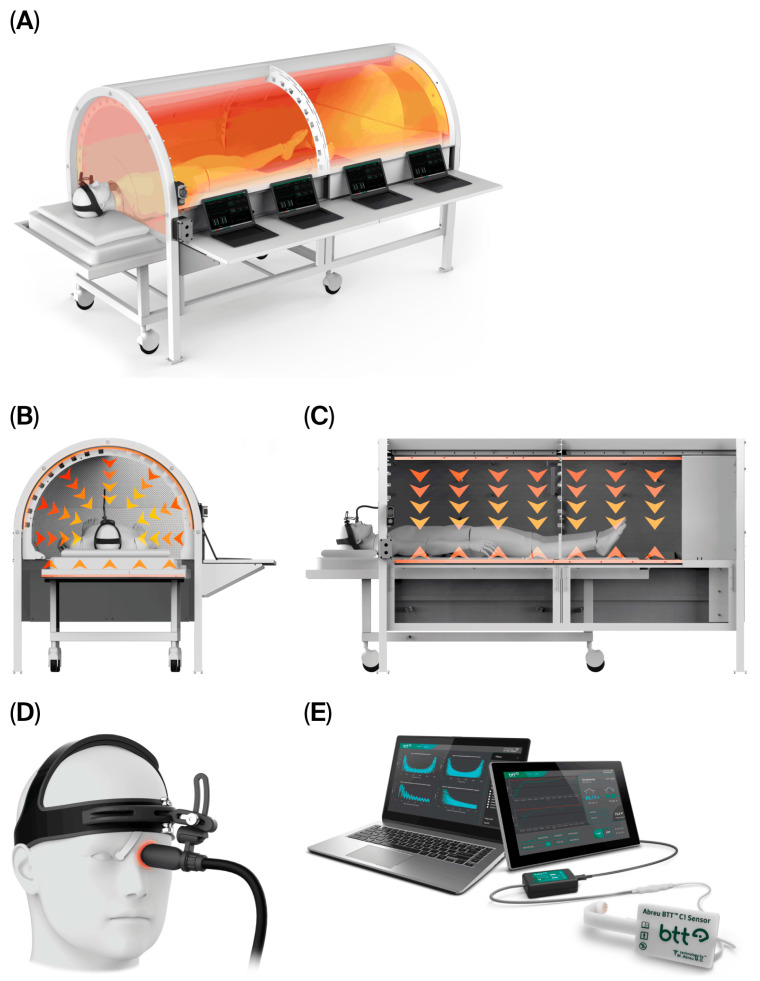
Schematic representation of the structural and functional components of the CBIT^2^ system, a computerized, intelligent thermal delivery system integrating radiative–conductive delivery and hypothalamic feedback modulation. (**A**) Modular structure of the BTT radiant heat chamber, with integrated segments that enable precise control of heat distribution across the body. (**B**) Frontal and (**C**) lateral views of the CBIT^2^ chamber, illustrating heat transfer pathways, with downward arrows indicating radiative heat flow and upward arrows indicating conductive heat flow (single up arrow). (**D**) BTT sensor assembly with an eyelid-mounted BTT thermal inductor. (**E**) BTT computerized platform using the FDA-approved Abreu-BTT 700 System (Brain Tunnelgenix Technologies Corp, Aventura, Florida, USA), which integrates the BTT sensor assembly, CBIT^2^ chamber, and an eyelid-mounted BTT thermal inductor. BTT: Brain–Eyelid Thermoregulatory Tunnel; CBIT^2^: Computerized Brain-Guided Intelligent Thermofebrile Therapy.

Following a comprehensive explanation of CBIT^2^, including its potential risks and benefits, the patient’s written informed consent was obtained. The procedure was conducted in accordance with U.S. FDA regulations, utilizing an FDA-approved computerized platform. The patient received CBIT^2^, a dual-modality heat delivery therapy that noninvasively induces therapeutic fever through programmed radiant and conductive delivery of heat to the skin, integrated with hypothalamic neuroregulatory feedback. The treatment was administered using the FDA-approved Abreu-BTT 700 System (Brain Tunnelgenix Technologies Corp, Aventura, FL, USA), and includes the BTT sensor assembly, a BTT radiant-heat chamber, and an eyelid-mounted BTT thermal inductor (see the schematic in Figure 1).

Central to the CBIT^2^ is the Thermofebrile Rhythm Engineering Protocol, which transforms brain temperature from a static measurement into a continuously monitored, dynamic pattern that is algorithmically recognized and modeled on the thermal rhythm of malarial fever [11,12,13,14,15,16,17,18,19,20,49,50,51,52], which is then reinterpreted within a controlled, brain-mediated, noninfectious, and precision-guided algorithm. CBIT^2^ digitally reengineers and condenses the malaria fever-based therapy first introduced by Wagner-Jauregg preserving its therapeutic objectives and cyclical dynamics while enabling safe, precise, and noninvasive brain-guided therapy.

CBIT^2^ reproduces the natural pattern of malarial fever through a controlled three-phase thermofebrile cycle—cold, hot, and sweating—modeled after the cyclical dynamics of Nobel Prize-winning malariotherapy. Each treatment preferably includes two consecutive thermofebrile cycles designed to follow the febrile rhythm of *Plasmodium vivax*-induced febrile paroxysms.

Thermofebrile Cycle Condensation and Dual-Phase Design: Classical malarial fever unfold over three distinct stages lasting 8 to 12 h: a cold stage lasting 1 to 2 h; a hot stage of approximately 3 to 4 h, reaching peak temperatures of up to 41.6 °C; and a sweating stage lasting 2 to 4 h [49,50,51,52]. These stages correspond directly to the CBIT^2^ phases, which replicate the triphasic structure of natural fever, but in a condensed format, and in a noninfectious, algorithmically controlled manner. The CBIT^2^ protocol condenses the entire cycle into a tightly regulated session of approximately 2.5 to 5.5 h, with the duration customized to each patient’s thermoregulatory response profile, pre-treatment biomarkers, and individual clinical requirements. Historically, malarial fever cycles exhibit progressively more intense febrile episodes, representing the body’s adaptive thermoregulatory and immune reinforcement with each recurrence. By digitally replicating this escalating biological pattern in a controlled, infection-free manner, CBIT^2^ aligns therapeutic intervention with the body’s own evolutionary logic for fever-mediated repair and detoxification. From a thermophysical and thermal physiological perspective, the first fever cycle also enhances heat transfer along the BTT, stabilizing heat transfer dynamics between the superior ophthalmic vein and hypothalamus. Once this equilibrium is established, the second cycle achieves more uniform and efficient heat distribution, allowing deeper engagement of the hypothalamic and thermoregulatory process critical to systemic coordination of the heat shock response.

Accordingly, the CBIT^2^ therapeutic protocol incorporates two sequential thermofebrile cycles within a single treatment session, each reproducing the three fundamental phases of malarial fever—cold, hot, and sweating—through precise, computer-controlled thermoregulation. The use of a dual-cycle design, in which the second cycle delivers a higher thermal load and slightly longer duration than the first, is grounded in both biological precedent and thermodynamic optimization principles. From a neurobiological standpoint, the first fever cycle functions as a priming phase, initiating the heat shock response. The method is based on the hypothesis that the initial brain-guided programed fever triggers an initial heat shock response, but its effects are potentiated only after transcriptional activation of HSPs has begun. Thus, the first cycle prepares the neural and cellular environment, inducing mild stress that preconditions the system for greater thermal tolerance and resilience. The second fever cycle builds upon this molecular foundation, and once the heat shock response is active, the enhanced thermotolerance allows the application of a higher temperature and extended hot phase without triggering hypothalamic cooling reflexes. This second cycle serves to amplify HSP expression and consolidate the clearance of misfolded proteins, thereby maximizing therapeutic efficacy while maintaining safety. In this way, the sequence follows the principle of thermodynamic reinforcement represented by an initial adaptive exposure followed by a stronger consolidation phase. As noted above, this design reflects the physiological patterns observed in natural tertian malarial fever, on which CBIT^2^ is modeled. Clinically, the rationale for this progression is equally strong. Patients with ALS or other neurodegenerative disorders often present with compromised neuromuscular and respiratory reserve. Beginning with a moderate thermal challenge in the first cycle ensures safety and allows real-time assessment of cardiovascular, metabolic, and autonomic responses. Once stability is confirmed, the second, higher-load cycle can safely deliver the necessary thermal energy to achieve maximal therapeutic activation without risk of overheating.

In the present case, the second cycle delivered a modest but measurable increase in temperature and duration than the first, consistent with the patient’s improved tolerance and adaptive physiological response. We hypothesized that the incremental escalation is analogous to a dose–response optimization, ensuring sufficient heat shock activation to drive molecular changes. Together, these cycles, one preparatory and one consolidative, represent a digitally reengineered, noninfectious translation of Nobel-recognized malarial fever therapy, preserving its cyclical therapeutic essence.

Thermofebrile Cycle Timing and Structure in CBIT^2^: CBIT^2^ was delivered in two condensed thermofebrile cycles to emulate the natural triphasic structure of malarial fever while maintaining clinical effectiveness and safety. Each cycle reproduced the characteristic cold (ramp-up), hot (peak), and sweating (resolution) stages through digitally controlled modulation of heat flow under continuous monitoring of physiological parameters. For the first cycle, the cold stage, corresponding to the gradual ramp-up of baseline brain temperature, lasted approximately 90 min. This cold stage provided a progressive rise in thermal energy, allowing thermoregulatory adaptation, with the goal of reaching about 41 °C to 41.6 °C. The subsequent hot stage (peak phase) was maintained for 15 min after the first cycle to assure that cooling mechanisms were actively suppressed to prevent counter-regulatory cooling by hypothalamic thermoregulatory response. The sweating phase spanned approximately 120 min, allowing the essential and closely monitored controlled decline in brain temperature to baseline (37 °C), thus reproducing the natural defervescence observed in classical tertian malarial fever. To optimize therapeutic efficacy, a second thermofebrile cycle was applied within the same treatment session, as shown above.

The CBIT^2^ protocol induces therapeutic fever using a dual-source heating architecture that integrates both radiant and conductive thermal modalities. The treatment chamber employs far-infrared radiative panels to deliver surface-directed heat, while a temperature-regulated thermal cover applies heat directly to the skin. To ensure precise regulation of core and brain temperatures, the CBIT^2^ algorithm also continuously monitors the tympanic membrane temperature, and uses six peripheral surface sensors positioned at key anatomical sites. These multisite thermal inputs, including cutaneous responses, are processed in real time to anticipate hypothalamic counter-regulatory mechanisms, as reflected by sympathetic activation or inhibition resulting in the vasoconstriction or vasodilation of cutaneous blood vessels.

This closed-loop feedback system synchronizes thermal delivery with hypothalamic thermoregulatory activity via heat delivered through the BTT, enabling the safe and effective induction of therapeutic fever while modulating hypothalamus-driven cooling reflexes. Upon detection of peripheral vasodilation, which is interpreted as the potential onset of hypothalamus-mediated cooling reflexes, the algorithm responds by increasing hypothalamic stimulation through modulated heat delivery, promoting vasoconstriction. This dynamic adjustment enhances heat retention and maintains upward thermal momentum as the temperature climbs toward the target fever range. In this way, CBIT^2^ leverages the natural thermal effector loop to reinforce rather than oppose fever induction, resulting in the precise, sustained elevation of brain temperature without triggering counterproductive autonomic responses.

This bidirectional thermal feedback architecture, which combines far-infrared radiation with conduction-based skin heating, enables precise induction of therapeutic fever while prioritizing patient safety. As a result, CBIT^2^ achieves a sustained and regulated elevation in brain temperature within a narrow therapeutic window, preserving the physiological benefits of malarial fever without its associated risks or the need for anesthesia. Throughout the treatment, key physiological parameters were monitored, including blood pressure, heart rate, and oxygen saturation, in addition to blood glucose levels to ensure metabolic stability. Hydration was managed through intravenous infusion of sodium chloride or Ringer’s lactate. Maintained alertness was confirmed by repeatedly engaging the patient in conversation. All CBIT^2^ sessions were administered by the first author (MMA), who is a licensed medical doctor, in his private practice at the BTT Medical Institute in Aventura, Florida, USA, in compliance with the State of Florida and U.S. FDA regulations.

### 2.2. Timeline of Treatment Sessions and Objective Assessments

The patient underwent CBIT^2^ on 29 January 2025 and approximately two months later, on 19 March 2025. Clinical and molecular measurements were collected at defined intervals to capture both the acute and cumulative effects of CBIT^2^. Baseline values (pre1) were obtained one day prior to the first treatment. Follow-up measurements were taken 24 h and 48 h later, with the 48 h value designated post1 and the treatment effect calculated as post1–pre1. A second baseline (pre2) was recorded one day before the second treatment, approximately two months later, allowing for an evaluation of changes during the inter-treatment interval (pre2–post1). Measurements taken 24 h and 48 h after the second session yielded the value post2, which was used to assess both the effect of the second treatment (post2–pre2) and the cumulative impact of both sessions (post2–pre1).

Objective testing before (pre1 and pre2) and after (post1 and post2) included the following groupings: upper extremity muscle strength and function, lower extremity muscle strength, stability and balance, and oropharyngeal strength. The biomolecular markers evaluated are detailed below. To facilitate assessment of changes within and between phases, %∆ was determined for each parameter. Although all objective tests and measurements were performed before and after the first CBIT^2^ session, not all were performed before and after the second session (primarily due to scheduling limitations). In addition to the objective determinations, clinical assessments included reports written by the patient and her physical therapist.

## 3. Follow-Up and Outcomes

The patient was closely monitored throughout the treatment period and during the six-month follow-up phase (up to 30 September 2025) to assess both clinical and functional responses to the intervention. The results detailed below summarize the short- and medium-term effects observed after each treatment session and during the intersession interval.

### 3.1. Strength and Function of Upper Extremities

Quantitative assessments of upper extremity strength and function before and after two sessions of CBIT^2^ therapy are detailed in Table 1.

#### 3.1.1. Number of Left and Right Arm Curls

The number of repetitive left and right arm curls with 5 lb weights (shown in Videos S1 and S2) improved following the first CBIT^2^ session. Notably, left arm performance increased from 10 curls during pre1 to 30 curls during post1, representing a 200% improvement. Upon reassessment two months later, approximately half of the initial improvement persisted, with the patient performing 20 curls at pre2. The second CBIT^2^ caused further improvement, with post2–pre2 = 36 − 20 = 16 curls, such that %∆post2–pre2 = 80% and cumulative %∆post2–pre1 [100 × (36 − 10)/10] = 260% improvement.

Similarly, the right arm demonstrated improvement after each treatment. After the first treatment, the number of curls increased from 15 to 26 repetitions, representing a 73.3% gain (%∆post1–pre1). Upon reassessment two months later, only 23.1% of this improvement was lost (%∆pre2–pre1), indicating that more than three-fourths of the initial improvement persisted over the ensuing two months. The second treatment caused further improvement, with post2–pre2 = 34 − 20 = 14 curls, such that %∆post2–pre2 = 70%, with a cumulative improvement of 126.7% relative to baseline (%∆post2–pre1).

In addition, we retrospectively viewed counts during the first 30 s of the videos obtained before and after the first session in the context of average normal values (between 12 and 17 curls/30 s) for 60–79-year-old females during senior fitness testing. Prior to the first session, the patient completed 7 curls/30 s with the left arm using a 5 lb weight, which was below the normal range. This increased to 9 curls/30 s after treatment (post2–post1 = 2 curls/30 s), which constituted a 22.2% improvement (Table 1). The rate of the right arm curls increased from 8 curls/30 s to 9 curls/30 s; post2–post1 = 1 curl/30 s, which constituted an 11.1% improvement. Videos were not obtained for the second session.

#### 3.1.2. Sustained Palmar Holding of 1 lb Weight

The patient’s ability to hold a 1 lb weight with an extended left hand (palm facing upward) improved from 102 to 187 s, representing an 83.3% improvement (%∆post1–pre1; Video S3). Upon reassessment two months later, about 67.6% (%∆pre2–pre1) of the improvement persisted, with the patient performing 171 s at pre2. The second treatment caused further improvement, with post2–pre2 = 19 s, such that %∆post2–pre2 = 11.1%, and a cumulative gain of 88 s (post2–pre1), representing an 86.3% improvement (%∆post2–pre1).

The patient’s ability to hold a 1 lb weight with an extended right hand increased from 112 to 140 s after the first session, representing a 25.0% improvement (%∆post1–pre1). Upon reassessment two months later, none of the improvement persisted, and performance declined to 105 s at pre2, such that %∆pre2–pre1 = 6.3% reduction in function. The second treatment caused further improvement, with post2–pre2 = 110 s, resulting in a 104.8% gain (%∆post2–pre2) and a cumulative 92.0% (%∆post2–pre1) overall improvement.

#### 3.1.3. Sustained Pinching (Holding) of 2 lb Weight

The patient’s capacity to pinch and hold a 2 lb weight (tested for left arm only) increased from 96 to 136 s (%∆post1–pre1 = 41.7% improvement) after the first session (Video S4). Upon reassessment two months later, slightly less than one-third of the increase persisted: pre2 = 108 s, such that %∆pre2–pre1 = 12.5% improvement.

The second treatment caused further improvement, with post2–pre2 of 154 − 108 = 46 s, such that %∆post2–pre2 = 42.6% after the second session. The second treatment led to a cumulative post2–pre1 = 58 s and a %∆post2–pre1 = 60.4% overall improvement.

#### 3.1.4. Maintenance of Grip

Resistance in the fatigue of grip test (right arm only) increased from 30.1 to 37.4 dynamometer force units after the first session (%∆post1–pre1= 24.3% improvement). Upon reassessment two months later, 18.1% of this initial gain was maintained (%∆pre2–pre1), such that pre2 = 35.8 units. The second treatment caused further increase, with an additional 2.3 units (post2–pre2 = 38.1 − 35.8), such that %∆post2–pre2 = 6.4%, resulting in a cumulative gain of 26.6% (%∆post2–pre1) in fatigue resistance relative to baseline (for details, see Table 1).

### 3.2. Agility, Balance, and Gait in Lower Extremities

Table 2 presents the quantitative assessment of lower extremity agility, balance, and gait before and after two CBIT^2^ sessions.

#### 3.2.1. Sustained Seated Leg Raises

Seated leg raises with a 10 lb weight (Videos S5 and S6) demonstrated endurance time improvements from 129 to 187 s for the right leg (%∆post1–pre1 = 45% improvement) and from 223 to 300 s (34.5% improvement) for the left leg (Table 2). Assessments of leg raises were not performed before or after the second treatment.

#### 3.2.2. Ability to Turn in Bed

Prior to treatment, the patient required 63 s to turn from one side to the other in bed (Video S7, Table 2). Following treatment, the time required for turning decreased to 36 s, representing a 42.9% (%∆post1–pre1) improvement. A repeat assessment was not performed before or after the second treatment.

#### 3.2.3. Center of Pressure (COP) Assessment of Postural Sway

**Figure 2:**
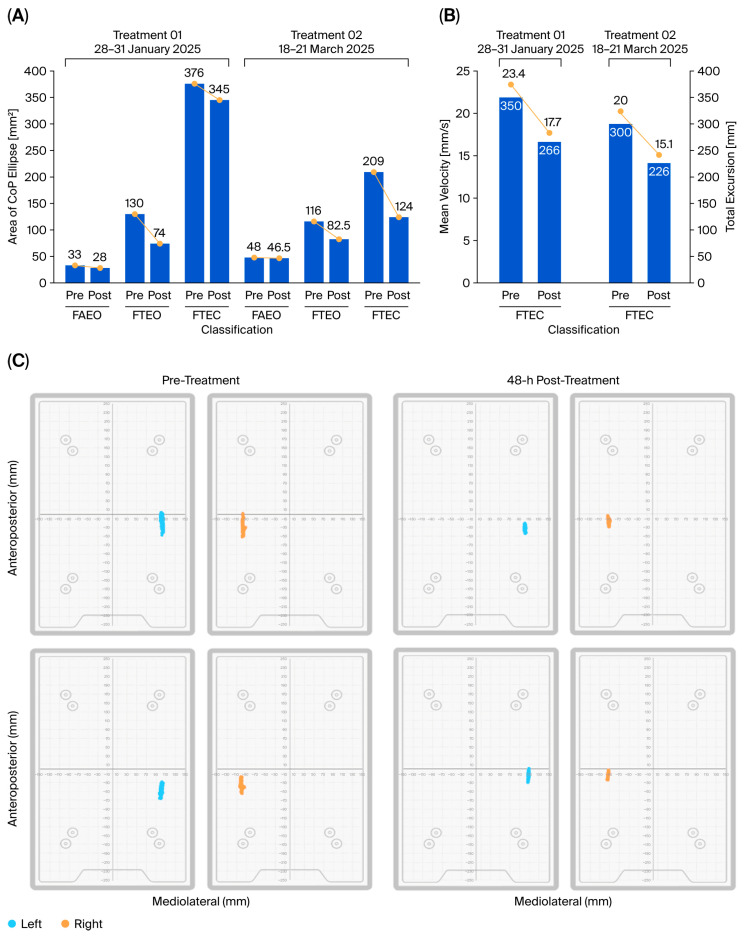
Postural stability and balance improvement following CBIT^2^ intervention with respect to the center of pressure (COP) at the point of application of the ground reaction force. (**A**) Cumulative bar graphs representing the area of COP ellipse (mm^2^) over increasing sensory and biomechanical challenges (FAEO, FTEO, FTEC), assessed pre- and post-treatment session. The figure demonstrates that, as the challenges in the test increase, the area of COP ellipse also increases. CBIT^2^ treatment reduces the area of COP ellipse, even under increased postural challenge (FTEC condition). (**B**) Total excursion (mm) and mean velocity (mm/s) of COP during session with FTEC, illustrating a reduction in both parameters. (**C**) Area of total excursion tracking on force deck (left (blue)/right (orange)) during the entire test duration of, pre-treatment and 48 h post-treatment, visually depicting the markedly reduced area of excursion in both sides after the first (upper panel) and second (lower panel) CBIT^2^ treatments. FAEO: feet apart and eyes open; FTEO: feet together with eyes open; FTEC: feet together with eyes closed; CBIT^2^: Computerized Brain-Guided Intelligent Thermofebrile Therapy.

A quiet stand test was conducted using the VALD Force Deck system to assess the patient’s stability and balance with respect to the center of pressure (COP) at the point of application of the ground reaction force. As summarized in Table 2, the total COP ellipse area, which is a marker of postural sway, was measured during quiet standing under three progressively challenging sensory and biomechanical conditions: feet apart and eyes open (FAEO), feet together with eyes open (FTEO), and feet together with eyes closed (FTEC). Our results revealed that the patient exhibited greater instability as sensory and biomechanical challenges increased, as evidenced by the progressive increase in COP ellipse area from FAEO to FTEC in the pre-treatment assessment (Figure 2A).

Baseline sway was greatest during FTEC due to the narrow base of support (FT) and the absence of visual input (EC), consistent with the patient’s history of positive Romberg tests. During FTEC, the first treatment was accompanied by a reduction in area of sway from 376 mm^2^ to 345 mm^2^, corresponding to an 8.2% (%∆post–pre1) improvement. Upon reassessment two months later, the patient evidenced greater improvement: pre2 = 209 mm^2^, a 44.4% improvement from baseline (%∆pre2–pre1). The second treatment caused further improvement during FTEC, as evidenced by a reduction in sway of 85 mm^2^ (post2–pre2), representing a 40.7% (%∆post2–pre2) gain and a cumulative overall improvement of 64.1% (%∆post2–pre1).

Changes during FAEO and FTEO are also reported in Table 2 and Figure 2A. As expected, these conditions revealed less initial compromise than FTEC. However, as calculated below, they showed greater improvements than the 8.2% decline in COP area observed in FTEC during the first CBIT^2^ treatment. During FAEO, the area of sway decreased from 33 mm^2^ to 28 mm^2^ (%∆post1–pre1 = −15.2%), and during FTEO, the area decreased from 130 mm^2^ to 74 mm^2^ (%∆post1–pre1 = −43.1%).

Considering the patient’s nystagmus and findings of visual–spatial dysfunction and improvement thereof (noted during the assessment, as reported below), it is not surprising that the first treatment had a greater impact on the combination of FT and EO as the treatment improved two forms of dysfunction [altered balance (FT) and visual–spatial dysfunction (EO)].

Additional values following the second treatment are provided in Table 2. Note that although FAEO decreased during each of the sessions, it increased in the interval between them, consistent with the inhibition of a progressive disorder after a single CBIT^2^ treatment, although the disorder persisted. The optimum number of CBIT^2^ sessions remains to be determined.

#### 3.2.4. COP Velocity and Excursion

As shown in Table 2, additional measurements during testing with FTEC showed that the CBIT^2^ reduced COP velocity and excursion (Figure 2B,C). After the first treatment, the mean COP velocity decreased from 23.4 mm/s to 17.7 mm/s, representing a 24.36% improvement. After both treatments, the mean COP velocity further decreased to 15.0 mm/s (with %∆post2–pre1 = 35.9% overall improvement).

With respect to the total excursion distance, the value decreased from 350 mm to 266 mm after the first treatment (24.0% improvement). After both treatments, the total excursion distance was reduced to 226 mm (with %∆post2–pre1 = 35.4% improvement).

#### 3.2.5. Gait

**Figure 3:**
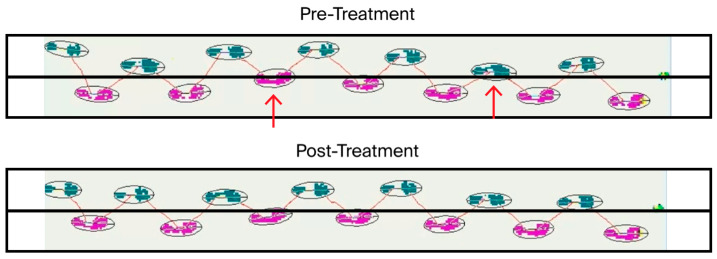
Gait analysis of straight-line walking before and after CBIT^2^ treatment. Prior to treatment, both the right foot (purple) and the left foot (green) crossed the midline (red arrows). Following treatment, gait restoration was evident, as neither foot crossed the center midline, indicating improved stability and alignment. CBIT^2^: Computerized Brain-Guided Intelligent Thermofebrile Therapy.

Gait measurement and analysis before and after the first treatment, conducted using the ProtoKinetics Zeno™ Walkway Gait Analysis System (Videos S8 and S9), provided further objective confirmation of underlying compromised stability and balance (Figure 3).

Measurements prior to the first treatment revealed that the patient’s step length, stride length, and gait speed were all below the range of normative data. As summarized in Table 2, the first CBIT^2^ treatment resulted in an increase in average step length from 24.56 cm pre1 to 29.73 cm post1, representing a 21.0% improvement; an increase in average stride length from 49.22 cm pre1 to 59.18 cm post1, indicating a 20.0% improvement; and an increase in gait speed from 0.41 m/s pre1 to 0.48 m/s post1, representing a 17.1% improvement.

As shown in Figure 3, the pretreatment gait analysis revealed significant lateral instability, characterized by pronounced swaying and deviation of both the right foot (pink trajectory) and left foot (green trajectory), which crossed the central line (indicated by arrows) during ambulation (Figure 3, upper panel). The post-treatment assessment demonstrated a marked improvement in dynamic balance, with the patient maintaining a stable, centered trajectory along a straight line, with neither foot crossing the central line (Figure 3, lower panel). Enhanced postural control and reduced lateral deviation were evident, reflecting improved muscular function, neuromuscular coordination, and gait symmetry following CBIT^2^. Gait analysis was not performed in association with the second CBIT^2^ session.

#### 3.2.6. Lower Extremity Agility

Additional lower extremity assessments revealed marked improvements in daily activities. Prior to treatment, the patient was unable to perform the cross-leg (ankle-to-knee) test (Video S10) or walk on her toes (Video S11). Following treatment, she was able to complete both the cross-leg test and the walk-on-toes test, indicating enhanced strength and motor coordination. Moreover, dorsiflexion, which had been challenging, became easier, allowing the patient to lift her feet higher with greater ease, approximately doubling her range of motion (Video S12).

### 3.3. Oropharyngeal and Pulmonary Assessments

#### 3.3.1. Muscles for Swallowing and Speech

Three Iowa Oral Performance Instrument (IOPI) parameters were assessed before and after the first CBIT^2^ session (Videos S13 to S15) to assess muscles essential to the abilities to swallow, prevent aspiration, and speak. As summarized in Table 3, anterior tongue endurance increased from 19 s to 58 s after the first session, corresponding to a 205.3% improvement. Posterior tongue endurance improved from 18 s to 30 s (%∆ = 66.7% improvement). Left-side lip strength increased from 20.2 kPa to 23.75 kPa (17.6% improvement). These findings suggest that CBIT^2^ significantly improved functions essential for swallowing, speech, and eating; this is consistent with reports provided by the patient and her caregiver in their written summary (below).

IOPI monitoring before and after the second CBIT^2^ session was limited to the assessment of posterior tongue endurance. Upon reassessment two months after the first treatment, posterior tongue endurance was 43 s, indicating a 43.3% (pre2–post1) increase during the intersession interval. The second treatment led to a decrease in posterior tongue endurance from 43 s to 33 s, representing a 23.3% decline (%∆post2–pre2). Overall, CBIT^2^ treatment resulted in a cumulative 83.3% improvement.

**Figure 4:**
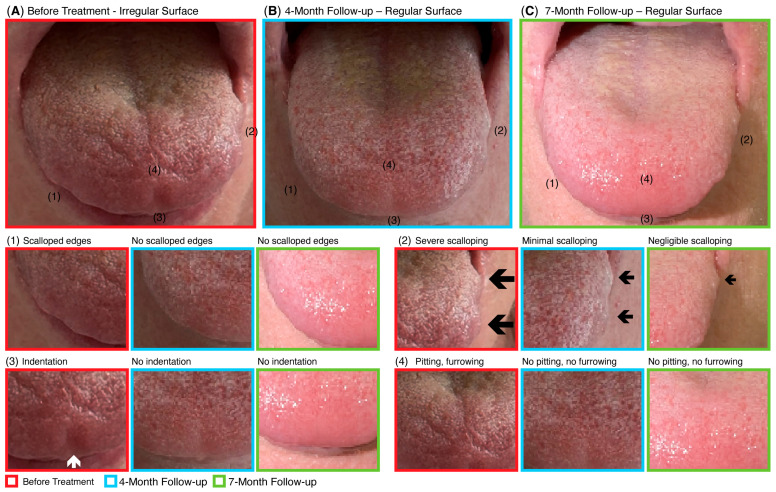
Progressive restoration of tongue morphology following CBIT^2^ treatment. Serial photographs demonstrate structural reconstitution of the tongue before treatment (**A**), at 4-month follow-up (**B**), and at 7-month follow-up (**C**), with magnified views of specific regions. At baseline, the tongue displayed irregular surface and characteristic features of atrophy seen in ALS, including (1) scalloped edges, (2) scalloping of the lateral border (black arrows; arrow size reflects the severity of the atrophy), (3) indentation (white arrow), and (4) pitting and furrowing. By 4 months post-treatment, a marked improvement was evident, with resolution of scalloped edges (1), absence of indentation (3), and disappearance of pitting and furrowing (4), while scalloping of the lateral border (2) had been reduced to minimal. At 7 months, these structural gains were maintained and further consolidated, with continued absence of scalloped edges, indentation, and pitting, and only minimal residual scalloping of the lateral borders. CBIT^2^: Computerized Brain-Guided Intelligent Thermofebrile Therapy.

Serial photographic documentation revealed progressive structural restoration of the tongue following CBIT^2^ treatment (Figure 4). At baseline (Figure 4A), the patient exhibited classical ALS-related atrophic features, including scalloped and irregular tongue borders, surface indentations, and pitting. By four months post-treatment (Figure 4B), these abnormalities showed marked resolution, with disappearance of pitting and indentations, and only minimal residual scalloping along the lateral borders. At seven months (Figure 4C), these improvements were maintained and further consolidated, indicating sustained tissue reconstitution and stability of the observed recovery. Such morphological normalization of tongue anatomy is consistent with the objective IOPI findings of enhanced anterior and posterior tongue endurance, suggesting better neuromuscular control essential for swallowing and speech.

#### 3.3.2. Pulmonary Function

Spirometry was performed in recognition of the progressive impact of ALS on respiration, most notably progressive life-threatening compromise of the diaphragm and intercostal muscles. Forced vital capacity (FVC), which is the volume of forced exhalation after a deep breath, and FEV1, which is volume exhaled as rapidly as possible in one second, were measured during pre1, post1, pre2, and post2 testing, and their ratio (FEV1/FVC%) was calculated (Table 4).

FVC, a metric commonly recommended for assessing ALS progression and severity because it reflects diaphragm and chest muscle strength, was 2.81 L upon initial baseline (pre1) spirometry. This value was below the lower boundary of ~3.2 L for normal FVC in subjects in the same sex and age group as the participant, but it was fortunately above the ~80% cutoff commonly considered ominous in ALS. An improvement in FVC after the first treatment was evidenced by an increase to 3.12 L at post1 (an 11.3% improvement). Upon reassessment two months later, FVC was 2.96 L, indicating that approximately half of the initial improvement persisted (pre2–post1 = 2.96 − 3.12 = −0.16 L).

The second treatment showed a small increase in FVC, reaching 3.00 L. This increase corresponded to an additional 0.04 L (post2–pre2), representing a 1.4% (%∆post2–pre2) gain and a cumulative overall improvement of 6.8% (%∆post2–pre1) relative to baseline, as opposed to the typical progressive decline in this vital indicator of ALS severity.

As shown in Table 4, the change in FEV1 from 2.36 L at pre1 to 2.40 L at pre2 constituted an overall %∆ of 1.7%. The baseline value for FEV1/FVC% was 84.1%, which was within the typical range of 75% to 85%. The ratio decreased from 84.1% at pre1 to 80.2% at post2, representing an overall decline of 4.6%.

At the 5-month follow-up, comprehensive pulmonary function testing was conducted at Northwestern University, Chicago, IL, USA, which demonstrated sustained or further improved respiratory performance, as evidenced by increases in both FVC (from 2.81 L at baseline to 3.07 L at follow-up) and FEV1 (from 2.36 L at baseline to 2.53 L at follow-up) values. The FEV1/FVC ratio rose slightly from 80.2% (post2) to 82.0% and remained within normal physiological limits, indicating proportional preservation of volume and flow. Overall, these follow-up findings suggest that the respiratory improvements observed after CBIT^2^ were not only maintained but, for certain parameters, continued to improve several months after treatment.

The increase in pulmonary function attributable to CBIT^2^ was consistent with reversal of the restrictive nature of ALS-induced muscle weakening as opposed to changes in airway tone.

### 3.4. Cognitive Function

**Figure 5:**
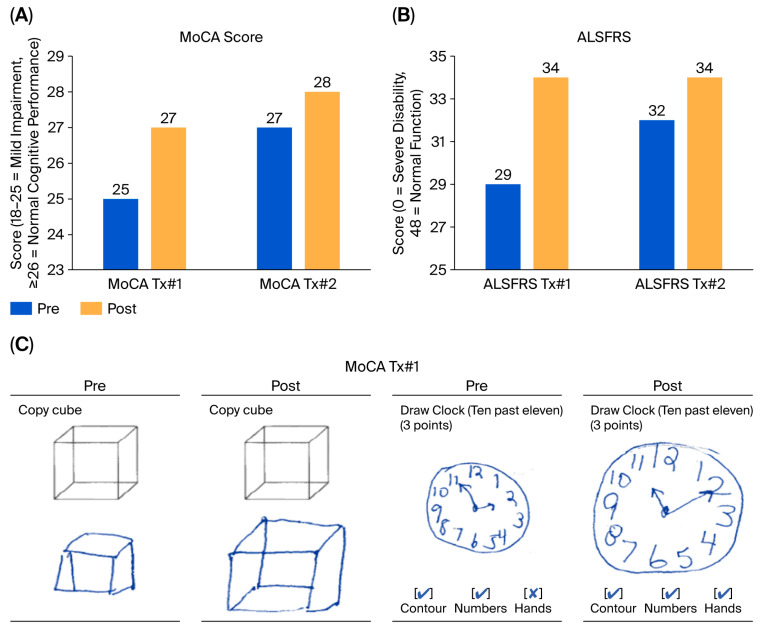
Cognitive and functional improvements following CBIT^2^ treatment, as assessed by the Montreal Cognitive Assessment (MoCA) and ALS Functional Rating Scale (ALSFRS). (**A**) Changes in MoCA total scores before and after two CBIT^2^ treatment sessions. In the first CBIT^2^ session, scores increased from 25 at pre-treatment assessment, consistent with mild cognitive impairment, to 27 at post-treatment assessment. In the second session, scores increased from 27 at pre-treatment assessment to 28 at post-treatment assessment, indicating normalization and subsequent incremental improvement of overall cognitive performance. (**B**) Changes in ALSFRS scores also improved progressively across the two treatment sessions, indicating enhanced and maintained overall functional performance. (**C**) The figure illustrates an improvement in the ability of the brain to process and interpret visual information in relation to space following CBIT^2^. Pre-treatment, the patient displayed geometric distortion in cube copying and incorrect clock hand placement, suggesting a visuospatial deficit. After treatment, the patient displayed appropriate structural and spatial accuracy and improved handwriting, with the resolution of handwriting resembling micrography previously evident in the clock-drawing and cube-copying tasks. ALS: amyotrophic lateral sclerosis; CBIT^2^: Computerized Brain-Guided Intelligent Thermofebrile Therapy; Tx#1: first treatment session; Tx#2: second treatment session.

Cognitive and functional evaluations were performed using the Montreal Cognitive Assessment (MoCA) and ALS Functional Rating Scale (ALSFRS), respectively (Figure 5A,B). MoCA scores demonstrated an improvement from mild cognitive impairment (score of 25) to normal cognitive function (score above 27) after the first treatment, indicating cognitive restoration (Figure 5C). Prior to treatment, the patient exhibited deficits in visuospatial/executive function, as evidenced by difficulties in correctly positioning the hands of a clock (ten minutes past 11) and copying a cube (Figure 5C), as well as compromised recall (of five words). After the first treatment, the patient accurately drew the hands of the clock and the cube and successfully recalled all five words. After the second treatment, her score increased from 27 to 28 between pre2 and post2. The maintained improvement between post2 and pre1 was consistent with the patient’s self-reported improvement at the end of the inter-treatment interval.

### 3.5. ALS Functional Rating Scale Cumulative Score

The patient was also assessed according to the 12 functional measures across four domains (bulbar function, respiratory function, gross motor function, and fine motor function) of the ALSFRS. Speech, salivation, swallowing, handwriting, cutting food and handling utensils, dressing and hygiene, turning in bed, walking, climbing stairs, dyspnea, orthopnea, and respiratory insufficiency were each rated on a 0 (worst) to 4 (best) scale, such that the maximum score was 48. As assessed by the nurse performing the testing, the patient exhibited an ALSFRS score of 29 prior to the first CBIT^2^ session, which increased to 34 after the first session, representing a 17.2% improvement in functional capacity. The most notable gains were observed in the scores for speech, swallowing, eating, turning in bed, and walking. Pre2 and post2 ALSFRS assessments were performed and showed maintained functional gain.

### 3.6. Hearing Function

An assessment of the patient’s hearing with automated testing of auditory sensitivity (GSI AMTAS) showed recovery from sensorineural hearing loss that was affecting her left ear. The pre-treatment audiogram revealed that, although the right ear exhibited normal hearing, the left ear demonstrated mild sensorineural hearing loss. Following the first treatment, both ears were within normal limits.

### 3.7. Molecular Measurements

**Figure 6:**
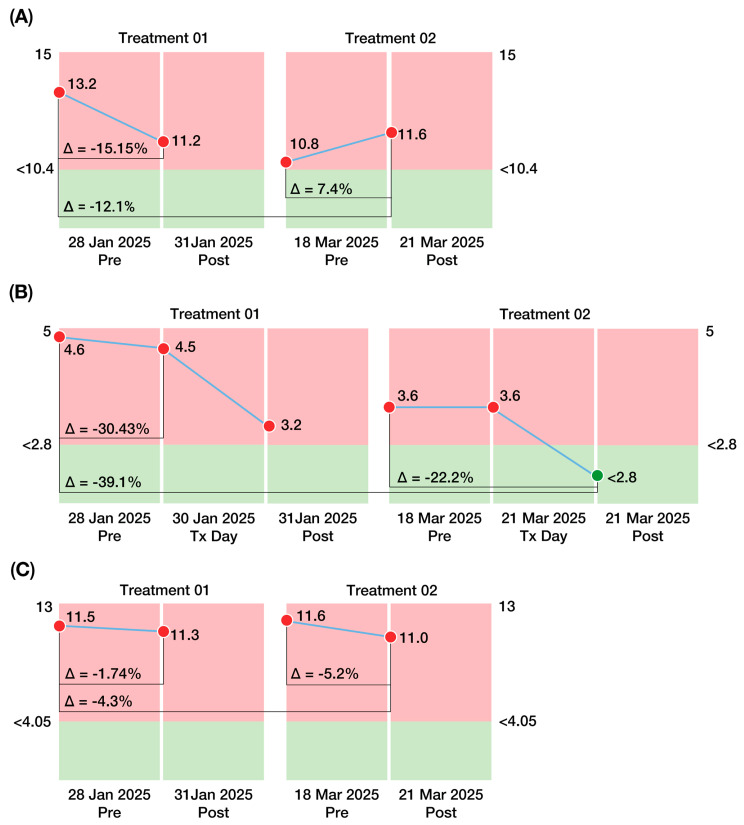
Changes in biomarkers associated with ALS severity before and after two CBIT^2^ treatment sessions. (**A**) Homocysteine levels demonstrated an overall downward trend across sessions, ranging from 13.20 µmol/L at baseline (pre-treatment) to 11.60 µmol/L after the second CBIT^2^ session, although values remained slightly above the reference range (<10.4 µmol/L). (**B**) Interleukin-10 (IL-10) levels showed progressive reductions, declining from 4.6 pg/mL at baseline (pre-treatment) to values within the reference range (<2.8 pg/mL) after the second CBIT^2^ session. (**C**) Neurofilament light chain (NfL) levels declined across both sessions, decreasing from 11.50 pg/mL at baseline (pre-treatment) to 11.00 pg/mL after the second CBIT^2^ session, although values remained above the reference range (<4.05 pg/mL). The figure also shows the variation (delta values) between the pre- and post-treatment measurements for both sessions, in addition to the cumulative difference from baseline to the post2 treatment session. Green: normal levels; Light Red: abnormal levels. ALS: amyotrophic lateral sclerosis; CBIT^2^: Computerized Brain-Guided Intelligent Thermofebrile Therapy; Tx Day: treatment day.

Biomarkers associated with ALS severity and a class of stress proteins were measured. All measurements were performed by independent third-party independent commercial laboratories in the United States. Changes in each biomarker related to ALS severity are shown in Figure 6.

#### 3.7.1. Homocysteine Levels

Homocysteine (normal plasma level < 10.4 µmol/L) decreased from 13.2 µmol/L to 11.2 µmol/L after the first treatment, such that a ∆post1–pre1 value of −2.0 µmol/L constituted a 15.2% improvement. The subsequent decline from post1 to pre2 of 0.4 µmol/L (a 3.6% decrease) indicated likely continuation of improvement during the two-month interval between treatments, such that the pre2 value was 18.2% lower than the baseline (pre1) measurement. After the second treatment, homocysteine levels increased by 0.8 µmol/L, indicating a 7.4% “worsening” during the second treatment. Nonetheless, the overall decline from pre1 to post2 (13.2 µmol/L to 11.6 µmol/L) constituted a 12.1% improvement (Figure 6A).

#### 3.7.2. Interleukin 10 (IL-10) Levels

IL-10 (normal serum level < 2.8 pg/mL) decreased from 4.6 pg/mL to 3.2 pg/mL after the first treatment, representing a 30.4% improvement (∆post1–pre1) The slight increase (0.4 pg/mL) over the next two months (%∆pre1–post1 = 12.5%) showed that most (87.5%) of the initial improvement was maintained. After the second treatment, IL-10 decreased by 0.8 pg/mL, corresponding to a 22.2% improvement. The overall (post2–pre1) decline from 4.6 pg/mL to 2.8 pg/mL represented a 39.1% improvement and constituted a reduction in IL-10 to a normal value (Figure 6B). Thus, elevated IL-10, a marker of the inflammatory pathway leading to mortality in ALS, was downregulated post-CBIT^2^, suggesting a potential shift away from fatal progression for the patient.

#### 3.7.3. Neurofilament Light Chain (NfL) Levels

Neurofilament light chain levels (normal < 4.5 pg/mL) decreased from 11.5 pg/mL to 11.3 pg/mL after the first treatment (%∆post1–pre1 = −0.2 pg/mL constituted a 1.7% improvement). An increase of 0.3 pg/mL over the next two months (%∆pre2–post1 = 2.7%) indicated that the first treatment did not result in a persistent decline. However, after the second treatment, neurofilament levels decreased by 0.6 pg/mL, indicating a reduction of 5.6% during the second treatment. The overall (post2–pre1) decline from 11.5 pg/mL to 11.0 pg/mL represented a 4.3% improvement (Figure 6C).

#### 3.7.4. Heat Shock Protein 70 (HSP70) Levels

In addition to the biomarkers associated with ALS severity, levels of HSP70 were quantified to assess activation of the cellular stress response pathway targeted by CBIT^2^ therapy. The changes in HSP70 are summarized in Table 5.

Measurement of HSP70 (which has an inconsistent normal range) showed that it increased from 88 pg/mL to 94 pg/mL at 48 h after the first treatment; a post1 48 h-pre1 change of 6 pg/mL, corresponding to a 6.82% increase. An additional increase of 51 pg/mL over the two-month interval between sessions (%∆pre2–post1 = 54.3%) indicated that the first treatment induced a greater subsequent increase, which persisted for at least two months.

After the second treatment, HSP70 levels decreased by 10 pg/mL (post2–pre2), corresponding to a 6.9% regression during the second treatment. However, the overall difference from pre1 to post2 (47 pg/mL) constituted a 53.4% increase in HSP70 levels. There are multiple variations of this class of proteins, each of which may have a varying time course, potentially confounding assessment of HSP70 changes. In addition to 48 h HSP70 samples, 24 h samples were also obtained. The 24 h value after the second treatment was 126 pg/mL, representing a 13.1% decrease (%∆post2 24 h-pre2) compared to the pre2 value. Our findings revealed a peak increase of 64% observed two months after the first treatment session (pre2–pre1).

### 3.8. Electrophysiological Evaluation: Evidence of Cessation of Motor Neuron Death Following CBIT^2^

Five months after the first treatment (24 June 2025), electrophysiological evaluations were repeated by independent university-based neurology services. In Table 6, these outcomes are compared by the authors to the pretreatment examination values (25 October 2024) to evaluate changes in motor unit integrity and neuromuscular signaling. The EMG protocol was designed to detect motor neuron death and the reemergence of electrical activity that could signify reinnervation. In this way, the electrophysiological data provided a real-time window into the potential for neuronal restoration.

The pretreatment examination performed on 25 October 2024 (see Appendix A.1), revealed active denervation, disease progression, and ongoing neurodegeneration, as evidenced by the presence of fibrillations, fasciculations, with active and chronic denervation changes in muscles innervated by the L4–S1 roots. These results are consistent with active motor neuron loss and increased neuronal injury. In sharp contrast, results from the EMG conducted five months after the first CBIT^2^ session (Appendix A.2) demonstrated the presence of chronic reinnervation in the vastus lateralis, rectus femoris, and tongue, without any signs of active denervation, and, remarkably, with the absence of fibrillations and fasciculations. These findings indicate a positive therapeutic response, which is detailed in the Discussion.

### 3.9. Self-Reported Clinical Improvements

One month after the first treatment, the patient and her physical therapist reported marked improvements in multiple aspects of daily living that were impossible or unachievable before CBIT^2^. These included successfully engaging in challenging and strenuous activities and demonstrating restored endurance and balance. The patient was able to attend a wedding, where she danced for four hours. She also reported that she now walks more frequently and without relying on her walker. She can go up and down the stairs independently, even while carrying objects such as a book. Getting in and out of bed has become easier, as she can now sit up, roll over, and lift her legs without external assistance. Her ability to perform daily activities has also improved. She reported that it is easier to put on socks, open jars, and grip objects such as a water bottle, a task that was previously challenging due to weakness in her hands. Balance and coordination have improved, allowing her to vacuum, carry objects while walking, and reach for items in the kitchen without difficulty. Shoulder pain, which previously affected her ability to drive, has completely resolved, making driving comfortable again. The patient’s speech and swallowing also have shown remarkable progress. She now speaks with greater clarity and projection, with a noticeable reduction in slurring. Her ability to sing has returned, and she is able to sing in the shower and in the car. Her ability to chew and swallow food was restored. The patient also reported that she regained a greater level of independence in daily tasks, including dressing, eating, and mobility. Her food no longer needs to be cut by her husband; the patient can do it by herself. Remarkably, the patient stated that she kneeled at church, which she had not done in years.

The overall improvement in the patient’s quality of life is evident, as she can now participate in social activities, such as attending events and engaging in physical exercise, with increased confidence and energy.

#### From Paralysis to Playing Golf

Recovery in this case of ALS was not limited to incremental improvements in strength or gait but extended to the restoration of complex, high-level motor coordination. Within three months of initiating treatment, the patient advanced from relying on a walker to playing golf—an activity that requires balance, strength, timing, proprioception, and precise neuromuscular sequencing. One month after the second treatment, she not only performed coordinated swings but also successfully completed golf holes, demonstrating functional integration of motor recovery. This outcome cannot be explained by compensatory adaptation; rather, it signifies the restitution of underlying neuromuscular and cortical control. The ability to transition from paralysis to the golf course parallels the dramatic functional restorations once observed during malarial fever therapy, reinforcing that reversal—not palliation—of neurodegenerative pathology is possible through brain-guided, reengineered fever therapy.

### 3.10. Physical Therapy-Assessed Outcomes

The patient’s strength and stability during physical therapy evaluations have improved. She successfully performed bridges on an exercise ball without assistance, demonstrating improved core strength. Her gait is smoother, with reduced stiffness and improved coordination. Her tandem balance, floor transfers, and walking endurance have also improved. Physical therapy assessments in Illinois documented measurable progress in various motor functions, including an increase in tandem balance time, a reduction in the time needed for the Timed Up and Go (TUG) test, and improved gait speed. Her tandem balance test showed an increase in stability, with her right side improving from 23 to 30 s and her left side increasing from 8 to 12 s. The TUG test, i.e., a measurement of the time it takes to rise from a seated position, walk three meters, turn, walk back, and sit down, improved from 18 to 16 s, indicating better movement efficiency. Her gait speed slightly increased, and her straight leg raise (SLR) supine test demonstrated greater flexibility and range of motion. These objective data points reinforce the visible functional progress observed in her daily activities. Her neuromuscular control has improved, leading to more fluid movements and reduced stiffness. The results of physical therapy tests, conducted in Illinois one month after CBT^2^ treatment, confirmed measurable improvements consistent with the tests performed 48 h post treatment at the BTT Medical Institute in Florida, USA.

## 4. Discussion

This case report presents the first documented reversal of ALS, a disease long regarded as irreversible and uniformly fatal, achieved through the first application of therapeutic fever to ALS, resulting in neurological, structural, molecular, cognitive, and electrophysiological reversal of ALS that directly challenges the entrenched view of inexorable progression in ALS. These findings provide historic proof-of-principle that ALS pathology is, in fact, reversible. While this outcome marks an unprecedented turning point in our understanding of ALS, its broader significance must now be tested through rigorous, large-scale clinical trials to confirm reproducibility, durability, and therapeutic potential across the spectrum of misfolding protein disorders including other neurodegenerative diseases.

In 1917, Wagner-Jauregg’s case report showed that deliberate fever induction could reverse dementia paralytica, the first proof that fever held curative power for neuropsychiatric disease [16]. This became a breakthrough that was later confirmed by trials and honored with the 1927 Nobel Prize. More than a century later, in that same lineage, the present case report demonstrates that fever, now digitally reengineered and brain-guided through CBIT^2^, can safely restore brain function in ALS, a disease long considered irreparable and fatal.

The BTT-based intelligent programmed fever treatment (formally termed CBIT^2^) resulted in complete reversal of ALS and restoration of lost brain function. This unprecedented outcome stands in sharp contrast to prevailing expectations for ALS. The Mayo Clinic report, for example, emphasized the inexorable course of the disease, stressing that “progressive lower motor neuron denervation is unfortunately inevitable” and communicated to the patient a life expectancy of only three to five years. This statement underscored the long-established scientific consensus, grounded in decades of rigorous research, that, once degeneration begins in ALS, there is progressive motor neuron loss, an inevitable decline, and a life expectancy limited to only a few years. For generations, this has defined ALS as a death sentence, with no path back once denervation sets in. In this case, however, that inevitability was not only averted but replaced by documented electrophysiological evidence of reversal of denervation, with elimination of fibrillation and fasciculation, demonstrated by irrefutable objective testing with EMG, the gold standard for ALS diagnosis. What science long judged as inexorable decline is here replaced by recovery, proving that the presumed irreversibility of ALS can, in fact, be undone, echoing the Nobel-Prize-validated precedent of fever therapy, which, a century ago, restored brain function in dementia paralytica.

After her initial diagnosis at the Mayo Clinic in Minnesota, USA, the patient was followed in her hometown at Northwestern University, which is ranked among the top neurology centers in the United States, where the ALS diagnosis was independently confirmed; she was thus maintained on the FDA-approved drugs for treating ALS, namely riluzole and edaravone. Despite expert care at both of these prestigious neurological centers, the Mayo Clinic and Northwestern University, her neurological function declined relentlessly, in keeping with the scientifically established expectation of inevitable progression and death in ALS.

A turning point came when an acquaintance, who had himself been successfully treated at the BTT Medical Institute, urged the patient to seek care in Florida, USA. Following treatment with CBIT^2^ by Dr. M. Marc Abreu at the BTT Medical Institute, the patient underwent a follow-up evaluation at Northwestern University. The neurologist (not involved with CBIT^2^) who performed the post-treatment EMG at Northwestern confirmed the unexpected electrophysiological finding of complete absence of both fibrillation and fasciculation potentials, which had been clearly present before fever therapy (see Appendix A). This EMG finding provides objective evidence of complete cessation of denervation and absence of motor neuron degeneration, consistent with disease reversal. According to the same diagnostic standards that had originally established the diagnosis of ALS, the patient no longer meets criteria for ALS and is, by definition, free of the disease. These electrophysiological results were further corroborated by concurrent blood biomarker normalization and structural restoration of tongue anatomy, collectively reinforcing the evidence of functional and neuronal regeneration following CBIT^2^.

Considering this unprecedented finding, all of the patient’s ALS-specific medications were discontinued, which is an outcome virtually unimaginable for a disease long regarded as irreversible and fatal. Both riluzole and edaravone, prescribed exclusively for ALS, were stopped, reflecting the extraordinary fact that the patient no longer met the diagnostic criteria for ALS and underscoring the plausibility of cure through programmed fever therapy. That grim prognosis for ALS is no longer a reality: instead of being shackled to a death sentence, the patient here now faces life in abundance, free of ALS, able to dance and even play golf and pickleball, as seen in the videos provided in this report.

In addition to the EMG demonstrating that ALS was no longer present, biomarkers shifted toward recovery, with reductions in neurofilament light chain and homocysteine, normalization of IL-10, a cytokine whose elevation as it was observed in this patient correlates with increased mortality [62], in addition to a surge in HSP70 expression. Clinically, she advanced from walker dependence to restored gait, safe swallowing, strengthened respiration, improved speech, and fully normalized cognition. Most strikingly, she regained the ability to perform complex motor tasks once thought irretrievable, such as swimming, walking unaided onto a golf green and sinking consecutive putts as well as returning to active sports. Most notably, no fluctuations were detected in the multifaceted neuromuscular, clinical, biomolecular, and electrodiagnostic improvements; every parameter showed sustained benefit, with no evidence of worsening in any domain. Spontaneous recovery or fluctuation in ALS is exceedingly rare. In 2023, Crayle et al. analyzed a cohort of 6187 ALS patients and identified only 46 cases that met the criteria for transient “ALS reversal,” defined as any objective improvement in function. This represents less than 1% of all cases and underscores that spontaneous or sustained functional recovery in ALS is exceptionally uncommon [63]. Consequently, the magnitude and persistence of neurological restoration observed following CBIT^2^ are very unlikely to represent spontaneous fluctuation. Future studies with serial MRI and advanced neuroimaging will be essential to document the structural correlates of recovery observed in this case. However, MRI is not part of the formal diagnostic or monitoring criteria for ALS, as it primarily serves to exclude alternative causes of motor neuron dysfunction rather than to confirm or track ALS progression [64].

The findings reported herein reveal restoration of motor neuron function following BTT-based intelligent programmed fever treatment, which is an outcome that directly contradicts the presumed irreversibility of motor neuron loss. This transformation, once unimaginable, echoes a truth first acknowledged nearly a century ago, when the Nobel Prize Committee recognized malarial fever therapy as the first demonstration of fever’s curative power for neuropsychiatric disease. The exceptional ALS reversal achieved here honors the vision, extending it into the modern era through BTT-based brain-guided programmed fever therapy. Nearly a century later, that foundation identified by the Nobel Prize has been extended and reengineered through CBIT^2^, transforming a once-forgotten treatment into a modern, infection-free, computerized intelligent brain-guided fever therapy that culminated in full reversal of ALS, which resulted in the discontinuation of all ALS-specific pharmacologic treatment.

The febrile response was digitally induced using CBIT^2^, a novel, artificial intelligence-enhanced treatment that delivers therapeutic fever safely and precisely through synchronized thermoregulatory modulation using an FDA-approved computerized platform [21]. CBIT^2^ digitally reengineers the curative principle behind the 1927 Nobel-Prize-winning malarial fever therapy into a brain-guided, programmable intelligent treatment. This fully noninvasive and unique procedure proved exceptionally safe, with no adverse effects or complications observed during treatment, within the critical 48 h post-treatment window, or across the entire six-month follow-up, affirming both its safety and durability of therapeutic effect. However, although the treatment is entirely noninvasive, it is important to acknowledge that the risk profile remains undefined, as the present findings are based on a single patient described in a single case report.

By normalizing the inflammatory response, as evidenced by normalization of IL-10, and activating the neuroprotective heat shock response without pharmacologic agents or infectious stimuli, CBIT^2^ provides a molecularly grounded and clinically actionable strategy for the restoration of motor neurons in ALS, a disease historically defined by relentless progression, therapeutic failure, irreversibility, and fatal outcomes.

Malarial fever therapy, initially developed to combat spirochete infection in neurosyphilis through induced fever, earned the Nobel Prize in Medicine in 1927, though its impact on brain pathology was not understood at the time. Nevertheless, this fever-based intervention was rapidly validated across Europe for achieving what was once deemed impossible: the reversal of advanced neurological disease, with the restoration of both motor and cognitive function in patients with dementia paralytica [11,12,13,14,15,16,17,18,19,20]. This disease reversal by malarial fever therapy, which was later extensively documented in diverse populations worldwide, emptied asylums once filled with paralyzed patients facing inevitable death, and established, more than a century ago, that brain damage is, in fact, reversible, which is the principle supporting the restoration of brain function in ALS reported in this article. However, there is a major distinction in the proposed mechanisms. Restoration from malarial fever was historically attributed to the fever-induced death of *Treponema pallidum*. By contrast, Abreu hypothesized that fever itself may restore brain function by activating the heat shock response, which acts directly on misfolded proteins such as TDP-43, playing a key role in their clearance, as well documented in previous investigations [27,28].

CBIT^2^ represents a paradigm shift from symptom management to a disease-modifying therapy that transforms fever into a computer-controlled treatment guided by real-time feedback from the brain’s thermoregulatory center. We proposed that the mechanistic rationale for this unprecedented recovery from ALS lies in the hypothalamus-guided induction of the heat shock response. By directly addressing misfolded proteins such as TDP-43, the molecular driver of ALS pathology, we hypothesized that CBIT^2^ restores proteostasis and neuronal function via HSP induction. No measurements of TDP-43 aggregates were obtained in this case, as the primary objective was patient treatment rather than research. While TDP-43 was not measured here, consistent with the fact that it had not been monitored even by her university-based neurologists, we acknowledge that this is a limitation of the report, while noting that it reflects standard clinical care rather than research-focused nature of the intervention. Nonetheless, the role of HSPs in the clearance of misfolded proteins, including TDP-43, is well established in the scientific literature [27,28,29,30,31], supporting the hypothesis that CBIT^2^ achieves its therapeutic effect through HSP induction leading to TDP-43 aggregate clearance. Experimental investigations support this hypothesis by demonstrating the role of HSPs in facilitating TDP-43 clearance and preventing its aggregation [27,28,29,30,31]. Activation of the HSF-1-dependent heat shock response has been shown to promote TDP-43 clearance in cultured cells and mouse skeletal muscle [30]. Overexpression of HspA5 has been reported to rescue TDP-43-induced toxicity in Drosophila models [29], and Hsp70 has been shown to bind the aggregation-prone region of the TDP-43 low-complexity domain (LCD), maintaining it in a dynamic, liquid-like state and preventing its pathological liquid-to-solid transition [31]. Nevertheless, the specific contribution of TDP-43 clearance should be further clarified in future evaluations of the proposed therapy herein, and direct measurement of TDP-43 should be incorporated into subsequent studies.

While mechanistic validation was not among the objectives of this case report, where the intervention was undertaken at the patient’s request for a fatal and otherwise untreatable condition, the findings of total reversal, as evidenced by the patient no longer meeting diagnostic criteria for ALS, underscore the importance of examining potential mechanisms consistent with the principles of mechanistic validation. Hartung et al. [65] described mechanistic validation in toxicology using an analogy that also applies to this therapeutic context: “At the first (phenomenological) level, we understand that our car (model) drove from city A (exposure) to city B (hazard manifestation), but we do not know which route it took. At the mode-of-action level, we understand the route. At the next (mechanistic) level, we see the complexity of interfering events. At the systems level, we model the dynamics of fluxes, roadblocks, deviations, and counter-regulatory events.” Applying this framework, the attempted treatment of a patient with ALS (city A) led to full disease resolution (city B). We therefore propose a mechanistic hypothesis linking CBIT^2^ to reversal of neurodegeneration through activation of the heat shock response, induction of HSPs, and consequent restoration of proteostasis. Experimental studies provide strong biological support for this mechanistic route: the heat shock response facilitates TDP-43 clearance and prevents its aggregation [27,28,29,30,31].

Although the mechanism cannot be proven in a single patient, these findings support a hypothesis of mechanistic validation involving HSP-mediated proteostasis restoration, reduction in protein toxicity, and clearance of misfolded TDP-43. The combination of neurological recovery, anatomical reconstitution of the tongue, biomarker normalization, and complete disappearance of denervation following CBIT^2^ is consistent with the mechanistic validation hypothesis for the induction of HSP leading to clearance of TDP-43 and neuronal restoration. In neurodegenerative diseases such as ALS, mechanistic validation serves to demonstrate that a therapy may target the underlying pathology rather than merely providing symptomatic benefit. Since misfolded proteins drive ALS pathology, the induction of HSPs provides a plausible mechanistic bridge between fever-based thermoregulation and disease reversal by reactivating the brain’s intrinsic molecular repair systems. Beyond documenting clinical recovery and total reversal of ALS, this case therefore contributes a hypothesis-driven framework for understanding the biological route of recovery, consistent with the observed increase in HSP70 and parallel electrophysiological, structural, and molecular improvements. Future studies are warranted to confirm this mechanistic validation hypothesis.

Unlike saunas or conventional hyperthermia, which trigger heat-dissipating reflexes and oppose therapeutic temperature elevation, CBIT^2^ operates in synchrony with hypothalamic pathways via the Brain–Eyelid Thermoregulatory Tunnel, enabling safe, titrated induction of therapeutic fever without adverse effects. This noninvasive approach transforms the hypothalamus from an opponent into the driver of fever, overcoming physiological barriers that have limited prior thermal therapies.

Using continuous, noninvasive monitoring of brain temperature and thermodynamics at the eyelid via the BTT, this intelligent system modulates thermal delivery via a thermal chamber and an eyelid heat inductor to prevent counterproductive hypothalamic cooling mechanisms, maintaining alignment with thermoregulatory responses required to induce a therapeutic thermofebrile response. CBIT^2^ integrates algorithmically controlled and synchronized delivery of radiative and conductive heat directed by hypothalamic signals through a computerized platform with a sensor assembly, thermal chamber, eyelid heat inductor, and intelligent closed-loop feedback control (Figure 1). Bidirectional heat exchange in concert with the hypothalamus is achieved by a sensor at the BTT site, which captures efferent brain thermal signals while a heat inductor delivers afferent inputs, resulting in a controlled rise and fall in brain temperature. This fluctuation in temperature, which may reach up to 41.6 °C, replicates the natural cyclic rhythm of malarial fever [49,50,51,52] within a safe, noninfectious therapeutic architecture, which was enabled by the discovery and characterization of the BTT [55].

Molecular evidence unveils a surprising mechanistic bridge between the dramatic recoveries once seen with malarial fever therapy in 1927 and the remarkable restoration of motor neuron function observed in ALS following CBIT^2^ in 2025. Though separated by nearly a century and distinct in etiology, neurosyphilis and ALS show a striking pathological convergence at the molecular level, marked by the accumulation of misfolded proteins that form TDP-43 aggregates [47,48]. This shared molecular pathology reveals disrupted protein homeostasis as a disease mechanism in both neurosyphilis and ALS, suggesting that targeted fever-based modulation may be an effective treatment for restoring neural function. Just as malarial fever therapy reversed motor and cognitive decline in dementia paralytica, the digitally controlled, brain-guided programmed fever described herein restored lost motor and cognitive function in ALS, revealing a potential molecular-mechanism-based treatment spanning a century of clinical practice.

By eliminating the risks associated with malarial infection, including potentially fatal complications such as cerebral malaria caused by parasitic invasion of the brain, CBIT^2^ safely replicates the intensity and cyclic dynamics of malaria fever, which was once used to cure dementia paralytica, transforming this century-old therapy into a noninfectious, titratable, intelligent, computer-guided intervention. In a single 2.5 to 5.5 h treatment session, CBIT^2^ delivers an average of at least two cycles of thermofebrile activation mimicking malarial fever, consisting of hyperthermia via a chamber and fever induction via hypothalamic signaling at the BTT site. These cycles are dynamically calibrated to the patient’s individual hypothalamic thermal signal, thereby avoiding conflict with central thermoregulation while enabling safe induction of a programmed thermofebrile response. 

Hypothalamus-driven treatment by CBIT^2^ stands in stark contrast to saunas and conventional skin-heating methods—including infrared hyperthermia devices, resistive heating systems, radiant heat panels, far-infrared equipment, infrared therapy beds, heat blankets, and whole-body hyperthermia systems—which act at the skin surface. These approaches stimulate cutaneous thermoreceptors and trigger hypothalamic heat-dissipating reflexes, as in saunas, turning the brain into an opponent actively fighting against the very temperature elevation required for therapy. In effect, the brain deploys its thermal defenses as a barrier, activating cooling mechanisms and preventing the safe achievement and maintenance of the core and brain temperatures needed for the robust and safe activation of heat shock transcription factors. CBIT^2^ uniquely overcomes this physiological opposition, transforming the hypothalamus from an opponent into the driver of therapeutic fever.

Additionally, hyperthermia methods without synchronization with hypothalamic thermoregulation can subject patients to significant physiological distress, including dizziness, nausea, vomiting, respiratory compromise, and even loss of consciousness, severe brain thermal injury, and coma [66], as heat externally applied to the body surface conflicts with central thermal signaling. Unopposed hypothalamus-driven heat dissipation mechanisms counteract heat applied to the skin surface, as in conventional whole-body hyperthermia, requiring excessive heating to achieve therapeutic temperature levels, which may lead to complications such as heatstroke, respiratory arrest, and brain injury. Despite their associated physiological burden and significant safety risks, conventional whole-body hyperthermia methods frequently fail to achieve or sustain therapeutic fever temperatures, such as the 41.6 °C observed in malarial fever [11,12,13,14,15,16,17,18,19,20,49,50,51,52], primarily due to misalignment with hypothalamic thermoregulatory control. Efforts to mitigate these uncomfortable and potentially serious adverse effects through conventional anesthetics are similarly counterproductive, as these agents disturb thermoregulatory function and are well documented to induce intraoperative hypothermia [67], directly opposing the thermal increase necessary for effective heat shock response.

The use of whole-body hyperthermia for cancer treatment was recently reviewed by Abreu et al. [68]; however, prior methodologies have been constrained by serious safety concerns, including coma resulting from excessive heat exposure [66]. These drawbacks are compounded by reliance on rectal temperature monitoring [66], a technique known for over a century to inadequately reflect brain temperature [69,70,71,72,73], resulting in undetected cerebral thermal overload and potentially life-threatening complications. In sharp contrast, CBIT^2^ titrates perihypothalamic temperature, using digitally synchronized, hypothalamus-guided modulation to replicate the therapeutic dynamics of malarial fever without triggering defensive cooling responses, enabling safe, well-tolerated, and effective treatment.

By advancing the Nobel-Prize-recognized fever-inducing disease reversal approach with digital precision, CBIT^2^ offers a safe treatment that can potentially not only slow but also reverse neurodegenerative processes. In the ALS case presented, reversal was supported by absence of denervation in EMG findings, reconstitution of tongue structure, motor function restoration, reductions in disease-associated blood biomarkers, and increased expression of HSPs. We saw the restoration of functional independence and recovery of complex motor tasks, such as dancing, singing, swimming, and playing golf, including coordinated swings and successful hole completion; the ALS case discussed herein thus suggests the reversal of underlying neurodegenerative pathology, closely resembling the cure and total restoration of lost brain function achieved a century ago with malarial fever therapy [11,12,13,14,15,16,17,18,19,20].

A profound impact, with implications for all ALS patients, was revealed electrophysiologically (see Appendix A). Following CBIT^2^, EMG demonstrated the complete absence of denervation across all tested muscles, which is a remarkable finding given that continuous active denervation, which was observed prior to BTT-based programmed fever therapy, is a hallmark of ongoing motor neuron death in ALS. The disappearance of fibrillations and fasciculations that were both previously present provides irrefutable electrophysiological evidence that the pathological process of motor neuron degeneration had been abolished. Moreover, the widespread chronic reinnervation changes observed in the absence of denervation confirmed that compensatory mechanisms were active and proceeding without ongoing neuronal destruction. These electrophysiological results were paralleled by the normalization of disease-associated molecular biomarkers and objective neuromuscular recovery in addition to restoration of tongue muscle, further reinforcing the conclusion that the underlying neurodegenerative process had been reversed. These clinical, molecular, anatomic, and electrophysiological findings challenge the long-standing view of ALS as an irreversible and fatal condition, suggesting that CBIT^2^ may reactivate the therapeutic principle of malarial fever, which was first successfully applied to reverse dementia paralytica and now offers a path to structural and functional brain recovery in ALS.

CBIT^2^ induced significant clinical improvements; the findings were supported by objective quantitative measures as well as patient self-report and physical therapist evaluations. These included increased strength in the arms and legs, improved balance and gait, improved bulbar function (enhanced speech, salivation, and swallowing), improved fine motor activity (handwriting and cutting food), coordinated motor (playing golf and pickleball) and gross motor (walking, stair climbing) activity, and improved pulmonary capacity, as well as gains in visuospatial function and cognition. Objective assessments confirmed enhanced motor control balance with eyes open (EO) and eyes closed (EC), feet apart (FA) and feet together (FT) (Figure 2), normalized gait (Figure 3), restored cognition (Figure 5), increased tongue endurance, and improved speech articulation. These functional improvements were accompanied by a five-point increase in the ALSFRS that was sustained over a six-month period, an outcome of particular significance given that the ALSFRS is primarily designed to measure progressive decline, and a sustained increase or stabilization in score is considered both exceptional and clinically meaningful [74]. Notably, even edaravone, an approved drug known for its ability to slow ALS progression by preventing neuronal damage, has not been able to produce a significant difference in ALSFRS-R scores compared to placebo [75]. Although previous studies demonstrated that a subset of patients with early-stage ALS experienced a slower rate of functional decline with edaravone, they have failed to demonstrate any increase in ALSFRS-R scores [76], as documented in this report.

At the biomarker molecular level, the response observed following CBIT^2^ indicates a shift from active neurodegeneration towards neuronal preservation and recovery, reflected in meaningful changes in biomarkers associated with ALS progression and fatal outcome. Blood levels of NfL are considered robust predictors of ALS progression [77], and their reduction, as shown in this case following BTT-based programmed fever, has been explored as a promising therapeutic target with disease-modifying potential, as exemplified by tofersen, an antisense oligonucleotide recently approved for the treatment of ALS in adults with SOD1 (superoxide dismutase-1) gene mutations [78].

Our results also revealed a striking and unprecedented molecular finding involving the normalization of IL-10, a cytokine showing persistent elevation in ALS—a hallmark of relentless neuroinflammation and a harbinger of poor prognosis and mortality [62]. This cytokine normalization (IL-10) followed the second treatment and coincided with a marked increase in HSP70 expression, rising from 6.82% after the first session to 53.4% after the second session. Elevated IL-10 has been inversely correlated with survival in ALS, and its downregulation may signal a reduced inflammatory burden and a departure from a fatal disease trajectory following CBIT^2^. Together, the reductions in neurofilament and homocysteine, which are biomarkers associated with neuronal death [77] and poor prognosis in ALS [79], respectively, alongside the normalization of IL-10 provide early evidence that CBIT^2^ may halt the pathological course of ALS and initiate a process of functional restoration and molecular reversal.

The neuromuscular, molecular, anatomic, and electrophysiological normalization observed are consistent with a CBIT^2^-induced rise in HSPs that are molecular chaperones regulating protein folding, degradation, and cellular stress responses while preventing the accumulation of neurotoxic aggregates, which are functions that are critically impaired in ALS [80,81]. Among HSPs, HSP70 is especially notable for its neuroprotective and restorative roles, including counteracting misfolded protein toxicity, enhancing cellular resilience to oxidative stress, and facilitating neuronal repair [35,82,83]. The ability to use programmed fever to noninvasively induce HSP70 in a clinical setting is a clinically significant therapeutic achievement, which has long been considered unattainable, and provides a mechanistic foundation for the reversal of neurodegeneration documented in this case.

ALS reversal aligns with the principle of malarial fever therapy which, as we revealed here, may have acted at the molecular level by refolding misfolded TDP-43 proteins and eliminating toxic aggregates in dementia paralytica. Studies in ALS animal models have shown that exogenous HSP70 administration enhances neuromuscular function and prolongs survival [36,83]. These findings parallel those in the present case, which demonstrated improved neuromuscular performance accompanied by normalization of IL-10 levels, which is a biomarker shift associated with extended survival, as elevated IL-10 has been linked to increased mortality [62]. Upregulation via histone deacetylase inhibitors enhances motor neuron resistance to stress-induced damage [84], and regional differences in HSP expression and inflammation in ALS may influence disease progression [85], supporting the potential of HSP-modulating therapies, as shown here.

The collapse of the cellular stress response in ALS disrupts proteostasis, leading to the accumulation of misfolded proteins and neurotoxic aggregates. In contrast, CBIT^2^ induced a robust upregulation of HSPs, which may restore proteostatic balance by enhancing chaperone-mediated protein folding and promoting the clearance of toxic aggregates. These molecular effects are consistent with the known role of HSP70 in maintaining proteostasis and regulating neuroimmune signaling. By activating HSF1, HSP70 suppresses inflammatory cascades involving NF-κB (nuclear factor kappa B) and JNK (c-Jun N-terminal Kinase) [86], in addition to reducing the production of cytokines [87], modulating glial activity by inhibiting pro-inflammatory microglial phenotypes (M1) [88], enhancing anti-inflammatory responses, and reducing astrocyte reactivity [86]. This immunological modulation highlights a broader mechanism by which CBIT^2^ restores immune homeostasis and reverses the molecular hallmarks of ALS progression and mortality. The upregulation of endogenous HSPs during CBIT^2^ thus offers a promising avenue for ALS treatment and disease reversal, paralleling the outcomes of malarial fever therapy that achieved clinical cure in neurosyphilis despite structurally damaged brain tissue harboring misfolded TDP-43, which is also present in ALS, further supporting the plausibility of a cure for ALS.

Clinical studies have tested the pharmacological amplification of the heat shock response, such as with arimoclomol, a co-inducer that enhances HSP70 expression. However, despite its mechanistic rationale, arimoclomol failed to provide meaningful clinical benefit in ALS patients and was associated with adverse effects, including an increase in NfL levels of approximately 29% compared with placebo [45], which is in sharp contrast to the reduction in NfL observed after CBIT^2^.

We anticipate that the impact of CBIT^2^ on HSP levels will constitute a therapeutic breakthrough. In this two-session introduction of CBIT^2^, HSP70 increased by 53.4% between pre1 and post2 determinations, reaching a peak increase of 64% two months after the first treatment session. The observation that HSPs reached their highest level in our patient at the assessment two months after the first CBIT^2^ treatment is consistent with a multi-step process that includes treatment-induced release of HSF1 which, in turn, causes DNA-mediated synthesis of HSPs via the heat shock element gene. In vitro primary culture models of ALS have shown that activation of HSF1 enhances motor neuron survival, reducing protein aggregation and mitigating proteotoxic stress [89].

The prolonged therapeutic impact of CBIT^2^ observed in this case reinforces its biological plausibility and supports the durability of its effect. The heat shock response activated by CBIT^2^ is a well-established neuroprotective and restorative mechanism associated with reduced neuroinflammation, enhanced cellular resilience to oxidative stress, and decreased accumulation of misfolded proteins, which are hallmarks of neurodegenerative disease [35,82,83].

In apparent contrast to the known effects of hyperthermia on HSP activity [90], a thoughtfully designed study using a mouse model of ALS reported potential therapeutic effects of chronic intermittent mild whole-body hypothermia [91]. By inducing mild hypothermia through chronic intermittent cooling cycles, the investigators observed delayed disease onset, improved neuromuscular junction integrity, and prolonged survival in the mouse model of ALS [91]. They also demonstrated that hypothermia restored levels of HSP70 and other molecular chaperones, further supporting its neuroprotective effects in the mice [91]. Their findings further support the rationale for developing thermally based therapies, involving both fever and cooling, for neurodegeneration. It remains to be determined how temperature and thermodynamics impact the structural and functional deterioration observed in ALS. The ability to noninvasively assess brain temperature and hypothalamic responses via the BTT and its correlation with disease activity may help address longstanding questions in ALS pathophysiology. However, species-specific genetic differences, particularly those influencing synaptic connectivity and neural circuitry, likely contribute to the therapeutic effects of hypothermia observed in ALS mouse models [92]. Although such animal models provide valuable mechanistic information, it is well recognized that interspecies distinctions limit the direct translation of findings to human neurobiology [92].

The customization of brain-guided programmed fever to enhance both efficacy and safety, nearly a century after the implementation of Wagner-Jauregg’s revolutionary life-saving fever therapy, was enabled by the discovery of the BTT, the first identified thermal waveguide in the human body, which consists of previously unrecognized bidirectional, bilateral pathways conducting thermal signals between the hypothalamus and the eyelid [55]. The discovery of the BTT has enabled, for the first time, noninvasive access to the hypothalamic thermoregulatory center, facilitating continuous brain temperature monitoring via a noninvasive surface sensor placed on the eyelid, as confirmed by recent studies showing brain cooling during yawning [56]. Studies have shown noninvasive measurements of brain temperature via the BTT during neurosurgery and distinguished BTT temperature measurements from those obtained using neighboring skin and blood surrounding the brain (e.g., during open-heart surgery) and ipsilateral to unilateral seizures [55]. Afferent thermal input from the eyelid skin to the brain is supported by the presence of heat-sensitive coatings on the trigeminal nerve, which courses along the wall of the cavernous sinus [93], in the terminal segment of the BTT [55].

Recognition of the BTT has enabled the development of the computerized system presented herein, which allows for the precise modulation of both systemic and brain temperatures through cyclical increases and decreases in heat delivered to the body core via a thermal chamber and to the hypothalamic thermoregulatory center via a BTT thermal inductor positioned on the eyelid (Figure 1). During CBIT^2^, digital control of the thermal chamber and eyelid-mounted inductor facilitates the maintenance of stable thermoregulation and brain-to-core temperature differentials that align with the body’s natural fever threshold [11,12,13,14,15,16,17,18,19,20]. Building on this thermodynamic foundation, the CBIT^2^ protocol initiates treatment with a gentle priming cycle followed by a more robust consolidation cycle, mirroring the physiological escalation of natural malarial fever. This sequencing provides a biological rationale for progressive HSP induction: the first cycle activates the heat shock factor 1 (HSF1) pathway and triggers early HSP synthesis, while the second reinforces these molecular and neuronal responses under stabilized thermal conditions, promoting sustained HSP expression, enhanced TDP-43 clearance, and restored proteostasis [27,28,29,30,31]. Thus, CBIT^2^ condenses and modernizes the Nobel-Prize-recognized malarial fever therapy into a precisely controlled, infection-free, dual-cycle therapeutic sequence that harmonizes safety, precision, and efficacy through intelligent brain-guided thermoregulation.

The sustained upregulation of HSPs and accompanying biomarker normalization, absence of denervation on EMG, and reconstitution of tongue structure previously affected by ALS-related atrophy collectively support a disease-modifying effect consistent with reversal of ALS, with the patient now free of the diagnostic criteria for ALS. Despite consistent improvement without any fluctuation, the duration and durability of these benefits beyond the current six-month follow-up remain unknown. Long-term follow-up will therefore be essential to ensuring that ALS does not recur and to determine the reproducibility of these findings. Furthermore, this intervention was directed exclusively toward patient care rather than research design, and therefore no control group or placebo was applicable. The clinical trajectory observed post-treatment highlights the importance of future controlled investigations to evaluate the therapeutic contribution of CBIT^2^, as direct comparison with standard-of-care agents such as riluzole and edaravone was beyond the scope of this case report. However, a head-to-head comparison of CBIT^2^ with riluzole or edaravone is not directly informative because these interventions act through fundamentally different mechanisms. CBIT^2^ induces heat shock responses and upregulates HSPs to promote proteostasis and facilitate clearance of misfolded proteins such as TDP-43, thereby targeting the underlying pathogenesis of ALS. In contrast, riluzole reduces excitotoxicity primarily by inhibiting presynaptic glutamate release and modulating voltage-gated sodium channels [22,23], while edaravone acts as a free-radical scavenger to mitigate oxidative stress [75,76]. Given these distinct mechanisms and therapeutic objectives, CBIT^2^ should be regarded as a pathogenesis-oriented, brain-guided therapy aimed at the restoration and potential reversal of neurodegeneration. Further studies and large clinical trials are required to determine whether repeated CBIT^2^ sessions can achieve cure comparable to the results historically observed with malarial fever therapy.

As brain-guided programmed fever offers a biologically supported method to augment endogenous HSP expression, it may enhance the effect of pharmacological chaperones such as arimoclomol [84], supporting future studies on combining CBIT^2^ with arimoclomol therapy to address proteostasis restoration. Moreover, through targeted induction of the heat shock response, CBIT^2^ may provide a biologically complementary mechanism to existing FDA-approved ALS therapies and potentially enhance their therapeutic efficacy. Riluzole, which reduces glutamate-mediated excitotoxicity [22], may be potentiated by fever-induced HSP-mediated neuroprotection via protein stabilization and the attenuation of oxidative stress. Edaravone, an antioxidant that mitigates neuronal oxidative injury [76], may benefit from enhanced cellular resilience and free radical buffering induced by HSP-mediated programmed fever. For tofersen, an antisense oligonucleotide that reduces toxic SOD1 protein levels in ALS associated with SOD1 mutation [78], CBIT^2^ may act synergistically by facilitating the refolding and clearance of misfolded proteins. This combination may support multimodal disease modification by addressing excitotoxicity, oxidative stress, and proteostasis dysfunction, complementing CBIT^2^-induced heat shock protein activation as a unified strategy with pharmacological agents for progressing towards a cure for ALS.

EMG evidence of denervation is required for the diagnosis of ALS. Prior to CBIT^2^, EMG at the Mayo Clinic showed fibrillation and fasciculation potentials, confirming active denervation and lower motor neuron degeneration. After treatment with CBIT^2^, repeat EMG at Northwestern University showed the complete disappearance of these findings, indicating cessation of denervation and absence of neurodegeneration. The patient no longer met diagnostic criteria for ALS, and ALS-specific medications were discontinued. This transition provides direct, objective evidence of reversal of neurodegeneration, corroborated by concurrent clinical recovery, biomarker normalization, and anatomical restoration of the tongue. Moreover, this case provides evidence consistent with neuronal regeneration. Following treatment with CBIT^2^, repeat EMG demonstrated complete disappearance of fibrillation and fasciculation potentials, with concurrent signs of reinnervation and corresponding clinical recovery. The resolution of denervation, together with biomarker normalization and anatomical restoration of the previously atrophic tongue (Figure 4), constitutes direct and objective evidence consistent with neuronal regeneration. ALS characteristically progresses without reversal; therefore, these electrophysiological, molecular, and structural findings collectively represent a documented case of neuronal restoration following CBIT^2^. 

A placebo effect cannot account for the objective findings documented in this case. Placebo responses are generally confined to subjective or transient symptomatic changes and cannot plausibly explain the multimodal objective improvements observed following CBIT^2^. The normalization of disease-associated biomarkers, such as IL-10, a key molecular marker linked to ALS progression and reduced survival [62], provides biochemical evidence of genuine disease modification beyond patient perception or expectation. Moreover, the complete disappearance of denervation, which was clearly present prior to treatment and objectively verified through electrophysiological testing (EMG), indicates a physiological reversal of motor neuron degeneration which is an outcome not attributable to placebo effect. In addition, Figure 4 illustrates the restoration of tongue anatomy that previously exhibited ALS-related atrophy, including loss of tissue with indentations, pitting, and ridging. The reconstitution of tongue structure provides visible anatomical evidence consistent with neuronal regeneration, further reinforcing the biological basis of recovery. Taken together, these electrophysiological, biochemical, and anatomical findings extend well beyond what could reasonably be attributed to a placebo effect, instead supporting a true physiological and molecular reversal of neurodegeneration following CBIT^2^ treatment. By activating HSPs and restoring proteostatic equilibrium, the brain-guided programmed thermofebrile activation introduced in this report may represent a potential therapeutic strategy for reversing neurodegenerative pathology, through activation of the heat shock response. This approach is hypothesized to act at the molecular root of neurodegeneration by facilitating the refolding or clearance of misfolded proteins, such as TDP-43 [27,28,29,30,31]. What was once a mysterious clinical phenomenon that restored function and completely reversed disease in patients with dementia paralytica over 100 years ago may be now be potentially reinterpreted as an early manifestation of a biologically grounded, HSP-mediated mechanism targeting misfolded TDP-43, rekindling the long-dormant legacy of fever-based treatment not as a historical curiosity but as a renewed frontier in modern molecular medicine.

Beyond ALS, this HSP induction breakthrough may open paths to treating and potentially curing other devastating neurodegenerative diseases, including Alzheimer’s disease, Parkinson’s disease, Huntington’s disease, Lewy body dementia, spinocerebellar ataxias, frontotemporal dementia, vascular dementia, multiple system atrophy, progressive supranuclear palsy, corticobasal degeneration, and prion diseases such as Creutzfeldt–Jakob disease, which are conditions long considered irreversible. In addition, the same mechanistic principles of reversing protein misfolding may extend to nonfatal but highly debilitating disorders, such as autism spectrum disorder [94,95,96] and diabetic neuropathy [97,98], widening the therapeutic horizon of brain-guided programmed fever.

This renewed molecular understanding of fever-induced therapeutic effects in neurological disease resulting in brain restoration emerges at a critical moment in history. ALS, long regarded as irreversible and fatal, may now serve as a gateway for therapeutic innovation for effective neurological treatment amid one of the most urgent global health crises, as highlighted by the WHO’s alarming announcement that neurological disorders affect over three billion people worldwide [2]. Moreover, according to the WHO, neurological disorders are the leading cause of disability [2], posing a profound threat to societal stability by diminishing workforce capacity, increasing long-term care demands, and straining healthcare systems worldwide. In the United States alone, Alzheimer’s disease generated USD 360 billion in direct costs in 2024 [99], an amount projected to reach USD 384 billion in 2025 [100], with an additional USD 413 billion linked to 19 billion hours of unpaid caregiving [100]. This totals an annual burden of USD 773 billion, reveals a looming global socioeconomic crisis. As WHO Director-General Dr. Tedros A. Ghebreyesus warned, “Neurological conditions cause great suffering to the individuals and families, they rob communities and economies of human capital” [2].

Neurological disorders now constitute one of the most debilitating and economically destabilizing health burdens worldwide, as they affect cognition, motor control, speech, and even vital functions such as swallowing and respiration. A central driver of this crisis is the pathological accumulation of misfolded proteins across multiple age groups, disrupting brain function in conditions ranging from ALS in otherwise healthy individuals and elite athletes to Alzheimer’s and Parkinson’s diseases in the aging population. The global wave of protein misfolding disorders extends even to young populations in the form of autism spectrum disorder, which is increasingly being recognized to involve disrupted proteostasis [94]; it may be responsive to therapies that activate HSPs [95]. The well-documented ‘fever effect’, in which febrile illness transiently improves behavioral symptoms in autism spectrum disorder, suggests that endogenous fever pathways can modulate neurological function [96]. This effect is further supported by studies demonstrating that sulforaphane improves autism spectrum disorder symptoms through activation of the heat shock response and upregulation of HSPs [95]. The WHO’s warning states that diabetic neuropathy is one of the other key conditions contributing to this alarming global neurological challenge [2], and protein misfolding is increasingly recognized as a fundamental mechanism in the pathogenesis of diabetic complications. In type 2 diabetes, amylin aggregates into toxic oligomers and fibrils within pancreatic islets, contributing to β cell dysfunction and metabolic decline [97]. New evidence indicates that HSP, particularly HSP70, can counteract this proteotoxicity. In animal models, induction of HSP70 via the small molecule KU 32 restored mitochondrial bioenergetics and reversed sensory neuropathy, offering a neuroprotective effect that addresses the misfolding pathology directly [98].

Misfolding protein disorders are increasingly linked to this global public health emergency, as neurological diseases now affect more than one in three people worldwide. With the WHO projecting that neurological disorders will become the second leading cause of death globally, the present disability crisis is on track to become a mass mortality event [4]. This escalating threat prompts a renewed scientific focus on therapeutic fever, which was first documented in a 1917 case report describing recovery from dementia paralytica [16]. Now, 108 years later, this case report on ALS may serve as a modern, molecularly based counterpart, reopening the possibility that advanced neurodegeneration can be therapeutically reversed. However, unlike the localized threat of neurosyphilis a century ago, the current challenge is global, as misfolded proteins are now recognized as central drivers of the most prevalent and debilitating neurological disorders.

The successful use of CBIT^2^ to induce therapeutic fever in ALS, a disease historically regarded as uniformly progressive and notably resistant to treatment due to its exceptionally high threshold for HSP induction [24], reframes this terminal condition as a potential therapeutic gateway. In this case report, the comprehensive restoration of motor and cognitive function following CBIT^2^ demonstrates that even the most treatment-refractory neuronal populations, such as motor neurons, can undergo recovery through the reactivation of endogenous repair mechanisms. If proteostatic resistance can be overcome in ALS, it is plausible that brain regions with lower activation thresholds may exhibit an even greater response. Indeed, preliminary findings from ongoing applications of CBIT^2^ in other misfolding-related disorders, including Alzheimer’s disease, Parkinson’s disease, Huntington’s disease, progressive supranuclear palsy, Lewy body dementia, and ataxias, have revealed clinical and biomarker changes suggestive of disease modification and brain restoration.

Our findings suggest that these therapeutic effects likely reflect the molecular reactivation of cytoprotective pathways, including HSP-mediated refolding of misfolded proteins such as TDP-43, restoration of proteostasis, and stabilization of neuronal structure and function. Together, these processes support the biological and molecular rationale for therapeutic fever as a strategy capable of restoring functional neural networks, opening new possibilities for care and redefining what is achievable in the treatment of neurodegenerative diseases. Collectively, these results position ALS not only as a clinical inflection point but as a critical first step in developing disease-modifying therapies and addressing the disease’s molecular roots, which span diverse neurodegenerative and neurodevelopmental pathologies such as autism spectrum disorder. Future studies involving independent replication across larger and more diverse cohorts will be essential to confirm and extend these preliminary observations. Such collaborative efforts will be critical to establish the generalizability, safety, and mechanistic validity of CBIT^2^ as a potential therapeutic approach for reversal of ALS.

### Patient Perspective

Following CBIT^2^, the patient’s functional gains continued to advance and remained strikingly evident. At a local golf course, three months after CBIT^2^, the patient walked unaided to the green with restored balance and motor control. She lined up her putt and sank the ball into the hole twice in succession. Further underscoring her recovery, she later shared a video of herself putting at home, sending the ball into a cup with a single, fluid swing on her first attempt (Video S16). She also returned to playing pickleball (Video S17) and to swimming in three different styles (Video S18). While visually impressive, these moments conveyed something beyond what any clinical scale, test, or biomarker could capture: the restoration of ordinary life and the freedom to live life to the fullest once more, echoing the profound and complete recoveries documented a century ago during malarial fever therapy. This regained ability to play golf, pickleball, and to swim revealed a clinically meaningful restoration of neuromotor precision, postural control, balance, and complex coordination, capacities once considered irretrievable in the course of ALS.

## 5. Conclusions

This case report demonstrates, for the first time, that ALS, long regarded as an irreversible and uniformly fatal neurodegenerative disorder, can be reversed through CBIT^2^, a fully noninvasive treatment that reengineers the 1927 Nobel Prize-recognized malarial fever therapy into a modern, intelligent, brain-guided digital intervention.

The patient, a 56-year-old woman, was diagnosed with ALS at the Mayo Clinic, based on EMG confirming denervation and neurological as well as MRI findings. A neurologist at Northwestern University examined the patient and confirmed the diagnosis of ALS, continuing the patient on the FDA-approved ALS drugs riluzole and edaravone. Despite expert management at these two highly ranked neurology institutions, her disease progressed relentlessly, consistent with expectations for ALS, a disorder defined by paralysis, respiratory failure, and death.

Following treatment by Dr. Marc Abreu in his private practice at the BTT Medical Institute in Aventura, Florida, USA, using CBIT^2^ delivered through an FDA-approved computerized platform, this fatal trajectory was not merely slowed but fundamentally transformed, achieving the neurological, molecular, structural, and electrophysiological reversal of ALS. EMG revealed the disappearance of fibrillation and fasciculations, signifying the cessation of motor neuron death while tongue structure was restored and biomarkers shifted toward recovery, with reductions in neurofilament light chain and homocysteine levels, normalization of IL-10 (with levels prior to CBIT^2^ linked to increased mortality), and a dramatic rise in HSP70 expression. Correspondingly, the patient progressed from walker dependence to restored gait, safe swallowing, improved respiration, enhanced speech, and cognition restored to a normal score. Most strikingly, she regained the ability to perform complex motor tasks, including walking unaided onto a golf green, sinking consecutive putts, playing pickleball, and swimming, signaling not only survival but a return to a full life once thought irretrievably lost. The absence of diagnostic evidence for ALS led to the discontinuation of all ALS-specific medications, an outcome previously unimaginable for ALS.

Dr. Wagner-Jauregg’s pioneering case report, published more than a century ago, described the reversal of dementia paralytica through deliberate fever induction [16], the first step in revealing fever’s curative potential for neuropsychiatric disease. Although the approach was initially met with skepticism, clinical trials confirmed its effects, reshaping neurology and psychiatry and earning Dr. Wagner-Jauregg recognition in the form of the 1927 Nobel Prize in Medicine. This report extends Wagner-Jauregg’s vision into the modern era with digital precision. With CBIT^2^, fever is no longer a dangerous byproduct of infection, but a brain-guided, programmable therapy delivered safely and noninvasively through the Brain–Eyelid Thermoregulatory Tunnel. Just as malarial fever once restored patients from the devastation of neurosyphilis, CBIT^2^ restored a patient from the devastation of ALS, a disease considered irreversible and fatal.

Notably, the patient in this case report presented with bulbar symptoms, including dysarthria and tongue atrophy, which are hallmarks of bulbar ALS. The appearance of bulbar dysfunction at any stage of ALS signals a shift to poorer prognosis and often rapidly fatal disease course [6,101,102,103]. Bulbar involvement, whether present at onset or developing later, is consistently associated with aggressive disease, accelerated progression, and reduced survival, which greatly impact quality of life due to compromise of swallowing, nutrition, and respiratory function. Following treatment with CBIT^2^, marked improvement in speech and reconstitution of tongue structure were observed, signifying restoration of bulbar motor function. Even bulbar ALS, which is one of the most devastating and aggressive forms of the disease, yielded to CBIT^2^’s precision-guided reawakening of neuronal function, which, against all odds, restored what had been clinically and biologically lost.

This convergence of digital medicine and neurobiology reawakens the Nobel-recognized principle that fever can restore neurological function, offering a molecularly grounded strategy for disease reversal, and correcting the 1927 original inaccurate assessments of the therapeutic mechanism consistent with fever acting on reduction or elimination of the pathogen causing syphilis. Unlike externally applied heat or standard hyperthermia, which may fail to trigger the heat shock response and may provoke important physiological distress due to high temperatures or potentially fatal complications [66], CBIT^2^ operates in synchrony with hypothalamic thermoregulation, enabling safe, titrated, and effective induction of therapeutic fever with robust HSP activation. This fully noninvasive procedure exhibited an excellent safety profile, with no adverse events observed during treatment, within the critical 48 h post-treatment window, or across six months of follow-up. However, although the treatment is noninvasive, it is important to acknowledge that the risk profile remains undefined, as the present findings are based on a single patient.

Beyond the confines of this case, our intervention responds to a broader global emergency, aligned with the Brain Economy Declaration at the June 2025 Brain Economy Summit [1], where G7 leaders urged brain health be prioritized as essential economic and societal infrastructure. In this context, the worldwide “Ice Bucket Challenge”, which was co-founded by ALS patient Pete Frates and joined by over 440 million participants globally, including virtually every major celebrity, prominent public figures, and many former heads of state, raised hundreds of millions of dollars for ALS research but has not yet produced a therapeutic breakthrough [104,105,106,107,108,109]. The loss of Frates to the same disease he sought to defeat underscores the urgent need to translate this global awareness into cures.

Cognitive impairment is now recognized as a common feature of ALS, with approximately 15–20% of patients developing progressive behavioral and cognitive changes consistent with frontotemporal dementia, reflecting degeneration of the frontal and temporal lobes [6]. In this case, the patient presented with early cognitive impairment (MoCA = 25) and achieved a normal score of 28 following two CBIT^2^ treatment cycles, accompanied by improved memory, attention, fluency, visuospatial, and executive function. These findings suggest that CBIT^2^ may extend beyond motor restoration to modulate cortical networks involved in cognitive dysfunction and dementia, raising the possibility that neurodegenerative cognitive decline in ALS, and potentially related dementias and Alzheimer’s disease, may be reversible via HSP induction.

These insights provide a compelling rationale for rigorous, large-scale clinical trials to confirm reproducibility, define durability, and establish the broader therapeutic potential of CBIT^2^. What begins here with ALS may extend to Alzheimer’s disease, Parkinson’s disease, Huntington’s disease, Lewy body dementia, spinocerebellar ataxias, frontotemporal dementia, multiple system atrophy, progressive supranuclear palsy, prion diseases, and beyond. The same molecular misfolding protein logic may also apply to nonfatal but debilitating disorders, such as autism spectrum disorder and diabetic neuropathy.

By reengineering the Nobel Prize-recognized fever therapy into a modern, digitally controlled, and infection-free platform, CBIT^2^ revives therapeutic fever after a century, not as memory but as method. Brain-guided intelligent programmed fever thus transforms a foundational medical discovery of the past into a potential new frontier for exploration by scientists and physicians worldwide, for restoring brain function and addressing the escalating global burden of neurological diseases. What is ultimately at stake is nothing less than the preservation of thought, memory, movement, and independence for more than one-third of humanity.

## Figures and Tables

**Table 1:** Quantitative evaluation of upper extremity strength and function before and after two CBIT^2^ sessions.

Upper Extremity Testing	1st CBIT^2^			2nd CBIT^2^			
	Pre1	Post1	%∆	Pre2	Post2	%∆	%∆Total ^a^
L arm curls (total #)	10.0	30.0	200.0%	20.0	36.0	80.0%	260.0%
R arm curls (total #)	15.0	26.0	73.3%	20.0	34.0	70.0%	126.7%
L arm curls (#/30 s)	7.0	9.0	22.2%				
R arm curls (#/30 s)	8.0	9.0	11.1%				
Sustained L palmar holding 1 lb weight (s)	102.0	187.0	83.3%	171.0	190.0	11.1%	86.3%
Sustained R palmar holding 1 lb weight (s)	112.0	140.0	25.0%	105.0	215.0	104.8%	92%
Sustained L hand pinching holding 2 lb weight (s)	96.0	136.0	41.7%	108.0	154.0	42.6%	60.4%
Fatigue of R arm grip (dynamometer units)	30.1	37.4	24.3%	35.8	38.1	6.4%	26.6%

^a^ Cumulative impact of both sessions (post2–pre1). L, left; s, seconds; R, right. lb, pound.

**Table 2:** Quantitative assessment of lower extremity agility, balance, and gait before and after two CBIT^2^ sessions.

Lower Extremity Testing	1st CBIT^2^			2nd CBIT^2^			
	Pre1	Post1	%∆	Pre2	Post2	%∆	%∆Total ^a^
Duration of R seated leg elevation with 10 lb weight (s)	129.0	187.0	45.0%				
Duration of L seated leg elevation with 10 lb weight (s)	223.0	300.0	34.5%				
Time required to turn in bed (s)	63.0	36.0	−42.9%				
COP area during FAEO (mm^2^)	33.0	28.0	−15.2%	48.0	46.5	−3.1%	40.9%
COP area during FTEO (mm^2^)	130.0	74.0	−43.1%	116.0	82.5	−28.9%	−36.6%
COP area during FTEC (mm^2^)	376.0	345.0	−8.2%	209.0	124.0	−40.7%	−64.1%
COP velocity of sway during FTEC (mm/s)	23.4	17.7	−24.4%	20.0	15.0	−25.0%	−35.9%
COP total excursion during FTEC (mm)	350.0	266.0	−24.0%	300.0	226.0	−4.7%	−35.4%
Average step length (cm)	24.56	29.73	21.05%				
Average stride length (cm)	49.33	59.18	19.97%				
Average gait speed (m/s)	0.41	0.48	17.07%				

^a^ Cumulative impact of both sessions (post2–pre1). COP, center of pressure; FAEO, feet apart and eyes open; FTEO, feet together with eyes open; FTEC, feet together with eyes closed; L, left; S, seconds; R, right; m, meter; mm, milimeter

**Table 3:** Comparison of oropharyngeal assessments before and after two CBIT^2^ sessions.

Oropharyngeal Parameters	1st CBIT^2^			2nd CBIT^2^			
	Pre1	Post1	%∆	Pre2	Post2	%∆	%∆Total ^a^
IOPI: anterior tongue endurance (s)	19.0	58.0	205.3%				
IOPI: posterior tongue endurance (s)	18.0	30.0	66.7%	43	33	−23.3%	83.3%
IOPI: left lip strength (kPa)	20.2	23.75	17.6%				

^a^ Cumulative impact of both sessions (post2–pre1). IOPI, Iowa Oral Performance Instrument.

**Table 4:** Pulmonary function assessments before and after two CBIT^2^ sessions and at 5-month follow-up.

Pulmonary Parameters	1st CBIT^2^			2nd CBIT^2^				
	Pre1	Post1	%∆	Pre2	Post2	%∆	%∆Total ^a^	Follow-Up
FVC (liters)	2.81	3.12	11.03%	2.96	3.00	1.4%	6.8%	3.07
FEV1 (liters)	2.36	2.44	3.39%	2.34	2.40	2.6%	1.7%	2.53
FEV1/FVC (%)	84.10	78.30	−6.90%	79.20	80.20	1.3%	−4.6%	82.0

^a^ Cumulative impact of both sessions (post2–pre1). FEV1, Forced Expiratory Volume in 1 s; FVC, forced vital capacity.

**Table 5:** Heat shock protein 70 (HSP70) levels before and after CBIT^2^ treatment.

Molecular Measurements	1st CBIT^2^				2nd CBIT^2^				
	Pre1	Post1 24 h	Post1 48 h	%∆	Pre2	Post2 24 h	Post2 48 h	%∆	%∆Total ^a^
HSP70 (pg/mL)	88.0	97.0	94.0	6.82%	145.0	126	135.0	−6.9%	53.4%

^a^ Cumulative impact of both sessions (post2–pre1). For %∆, post values were calculated using the 48 h values; HSP70: heat shock protein 70.

**Table 6:** Comparison of pre-treatment and post-treatment electromyography results.

Parameter	25 October 2024 (Mayo Clinic)	24 June 2025 (Northwestern University Hospital)
Fibrillations	Present = active denervation, motor neuron death	Absent = denervation abolished, no longer motor neuron death
Positive sharp waves	Present (implied)	Absent
Fasciculations	Present = motor neuron degeneration, dying lower motor neuron	Absent = motor neuron degeneration abolished, no longer lower motor neuron dying
Recruitment pattern	Reduced in multiple muscles	Full in most, mildly reduced in few
Reinnervation	Yes (chronic reinnervation)	Yes (chronic reinnervation)
Bulbar involvement	No active denervation	No active denervation
Nerve conduction	Normal	Normal
Overall Assessment	Active denervation consistent with progressive motor neuron disease marked by ongoing motor neuron death and dying lower motor neurons	No active denervation consistent with disease re-versal and effective therapeutic response

## Data Availability

The original contributions presented in this case report are included in this article/its Supplementary Material. Further inquiries can be directed to the corresponding author(s).

## Supplementary Materials

The following supporting information can be downloaded at: https://www.mdpi.com/article/10.3390/diseases13110371/s1, Video S1: Left arm curls with 5 lb weights; Video S2: Right arm curls with 5 lb weights; Video S3: Sustained palmar holding of 1 lb weight (left side); Video S4: Sustained pinching holding of 2 lb weight (left side); Video S5: Sustained seated leg raise elevation (right side); Video S6: Sustained seated leg raise elevation (left side); Video S7: Ability to turn in bed (before treatment); Video S8: Gait measurement before treatment; Video S9: Gait measurement after treatment; Video S10: Lower extremity agility (cross-leg) before treatment; Video S11: Lower extremity agility (walk on toes) before treatment; Video S12: Lower extremity agility (dorsiflexion) after treatment; Video S13: IOPI anterior tongue endurance value before and after the first CBIT^2^ session (close-up view); Video S14: IOPI posterior tongue endurance value before and after the first CBIT^2^ session (close-up view); Video S15: IOPI lip strength evaluation (left side); Video S16: Demonstration of the patient’s ability to play golf following CBIT^2^ treatment; Video S17: Demonstration of the patient’s ability to play pickleball following CBIT^2^ treatment; Video S18: Demonstration of the patient’s ability to swim following CBIT^2^ treatment.

## Abbreviations

The following abbreviations are used in this manuscript:

AI	Artificial intelligence
ALS	Amyotrophic lateral sclerosis
ALSFRS	Amyotrophic Lateral Sclerosis Functional Rating Scale
BTT	Brain–Eyelid Thermoregulatory Tunnel
CBIT^2^	Computerized Brain-Guided Intelligent Thermofebrile Therapy
CK	Creatine kinase
COP	Center of pressure
EMG	Electromyography
FAEO	Feet apart and eyes open
FDA	Food and Drug Administration
FEV1	Forced Expiratory Volume in 1 s
FLAIR	Fluid-attenuated inversion recovery
FTEC	Feet together with eyes closed
FTEO	Feet together with eyes open
FVC	Forced vital capacity
HSP	Heat shock protein
IL-10	Interleukin-10
IOPI	Iowa Oral Performance Instrument
IVIg	Intravenous immunoglobulin
JNK	c-Jun N-terminal kinase
MoCA	Montreal Cognitive Assessment
MRI	Magnetic Resonance Imaging
NF-κB	Nuclear factor kappa B
NfL	Neurofilament light chain
SLR	Straight leg raise
SOD1	Superoxide dismutase-1
SWI	Susceptibility-weighted imaging
TNF	Tumor necrosis factor
TSH	Thyroid-stimulating hormone
TUG	Timed Up and Go
WHO	World Health Organization

## Appendix A

### Appendix A.1

Appendix A.1 presents the electromyography report performed on 25 October 2024, at Mayo Clinic, Rochester, Minnesota, USA.

- DIAGNOSIS: ALS
- RESULT: DENERVATION WITH FIBRILLATION AND FASCICULATION PRESENT.

### Appendix A.2

Appendix A.2 presents the Electromyography report performed on 24 June 2025, at Northwestern Medicine NMH Neuro Testing CTR, Chicago, Illinois, USA, five months after the first treatment.

- DIAGNOSIS: ALS NO LONGER PRESENT
- RESULT: ABSENCE OF DENERVATION, FIBRILATTION AND FASCICULATION NO LONGER PRESENT.
